# Role of polygenic risk scores in the association between chronotype and health risk behaviors

**DOI:** 10.1186/s12888-023-05337-z

**Published:** 2023-12-20

**Authors:** Yi Zhang, Shuqin Li, Yang Xie, Wan Xiao, Huiqiong Xu, Zhengge Jin, Ruoyu Li, Yuhui Wan, Fangbiao Tao

**Affiliations:** 1 Department of Maternal, Child and Adolescent Health, School of Public Health, Anhui Medical University, No 81 Meishan Road, 230032 Hefei, Anhui China; 2 NHC Key Laboratory of Study on Abnormal Gametes and Reproductive Tract, No 81 Meishan Road, 230032 Hefei, Anhui China; 3 MOE Key Laboratory of Population Health Across Life Cycle, No 81 Meishan Road, 230032 Hefei, Anhui China

**Keywords:** Health risk behaviors, CLOCK-PRS, Chronotype, Gene-environment

## Abstract

**Background:**

This study explores the association between chronotypes and adolescent health risk behaviors (HRBs) by testing how genetic background moderates these associations and clarifies the influence of chronotypes and polygenic risk score (PRS) on adolescent HRBs.

**Methods:**

Using VOS-viewer software to select the corresponding data, this study used knowledge domain mapping to identify and develop the research direction with respect to adolescent risk factor type. Next, DNA samples from 264 students were collected for low-depth whole-genome sequencing. The sequencing detected HRB risk loci, 49 single nucleotide polymorphisms based to significant SNP. Subsequently, PRSs were assessed and divided into low, moderate, and high genetic risk according to the tertiles and chronotypes and interaction models were constructed to evaluate the association of interaction effect and clustering of adolescent HRBs. The chronotypes and the association between CLOCK-PRS and HRBs were examined to explore the association between chronotypes and mental health and circadian CLOCK-PRS and HRBs.

**Results:**

Four prominent areas were displayed by clustering information fields in network and density visualization modes in VOS-viewer. The total score of evening chronotypes correlated with high-level clustering of HRBs in adolescents, co-occurrence, and mental health, and the difference was statistically significant. After controlling covariates, the results remained consistent. Three-way interactions between chronotype, age, and mental health were observed, and the differences were statistically significant. CLOCK-PRS was constructed to identify genetic susceptibility to the clustering of HRBs. The interaction of evening chronotypes and high genetic risk CLOCK-PRS was positively correlated with high-level clustering of HRBs and HRB co-occurrence in adolescents, and the difference was statistically significant. The interaction between the sub-dimensions of evening chronotypes and the high genetic CLOCK-PRS risk correlated with the outcome of the clustering of HRBs and HRB co-occurrence.

**Conclusions:**

The interaction of PRS and chronotype and the HRBs in adolescents appear to have an association, and the three-way interaction between the CLOCK-PRS, chronotype, and mental health plays important roles for HRBs in adolescents.

**Supplementary Information:**

The online version contains supplementary material available at 10.1186/s12888-023-05337-z.

## Background

### Health risk behaviors

Adolescent health risk behaviors (HRBs) have received worldwide attention. Adolescent HRBs generally refers to behaviors that cause direct or indirect damage to adolescent health, happiness and quality of life [1]. Results from the 2017 National Youth Risk Behavior Survey (YRBS) from the United States indicate that many high school students engage in health risk behaviors that are associated with the leading causes of death among people aged 10–24 in the United States [2]. According to a recent study in Brazil that involved a large sample of adolescents, 8.8% of the adolescents did not have HRB; 34.5% had one; 42.7% had two; and 14.1% had three or four [3]. Other studies have shown that behaviors and lifestyles developed during adolescence are maintained into adulthood in the form of growth “trajectories” that affect health to varying degrees and may increase the risk of morbidity and mortality [4, 5]. The causes of HRB outcomes in adolescents are complex, and although most studies agree that HRBs is caused by multiple factors, including at the individual, family, or school level, these factors do not seem to fully explain the current high incidence of HRB. Therefore, it is necessary to identify other factors that contribute to the occurrence of HRB and consider applying the theories and/or concepts that drive these factors to the embodiment of multiple behavioral clusters.

### Chronotype

Circadian rhythm refers to the 24-hour physiological and behavioral rhythm of the individual, divided into endogenous and exogenous rhythms, when the two “mismatch” circadian rhythm may appear disorder. A person’s preference for getting up early or going to bed late is influenced by their genetic makeup [6]. Chronotype, or morning and evening preference, which is defined as the type of circadian rhythm of an individual, is also a manifestation of the biological characteristics that describe people’s sleep habits, usually determined by changes in sleep-wake cycles but also influenced by external environments, and is associated with adolescent HRBs [7]. Chronotype also refers to the time preference of individual sleep activities, which is generally divided into “morningness,” “intermediate,” and “eveningness”, it can be assessed by using a variety of methods, for example, the type of time an individual has in the morning or in the evening [8]. There is a sharp shift toward evening sleep patterns at the beginning of adolescence, a peak at the end of adolescence, and then a gradual shift toward morning sleep patterns as the aging process takes place [9]. This shift is influenced by the interaction of genetic, environmental and social factors with the adolescent development process. Other studies suggested that morningness is a protective factor for negative mood [10, 11]. A person’s chronotype can change with age and gender, morning chronotype mainly occurs in childhood and late adulthood, and generally transitions to evening chronotype during adolescence [12–14]. Before middle age, women also tend to have earlier chronotypes than men [15]. A study revealed that younger women showed a decrease in sleep duration and an increase in sleep deprivation, while older adults and men showed an increase in sleep duration [16], whereas another study showed that lower rMEQ scores were significantly associated with being male, young, and having higher scores on the Insomnia Severity Index and the Hospital Anxiety and Depression Scale [17]. Overall, study results demonstrate that sex differences in sleep patterns are different based on the social environments and population—a topic worthy of further discussion.

### Gene × environment

The idea that genetic or biological tendencies and environmental factors influence individual psychological behavior is widely accepted. Before a long period of early measures of specific genes, researchers proved that overall genetic effects (i.e., inheritance) may be due to different environmental factors [18]. At the beginning of the century, a study of gene × environment (G×E) identified functional polymorphisms in the serotonin transporter (5-HTT) gene promoter regions that modulate the effects of stressful life events on depression [19]. Individuals with one or two short allele polymorphisms of the 5-HTT promoter exhibited more depressive symptoms, diagnosable depression, and suicidal tendencies related to stressful life events than individuals with homozygous long alleles [19]. Such discoveries began to attract the attention of a wider range of research fields [20, 21]. In recent years, in addition to the influence of G×E interaction on mental health, some studies have found that the interaction influences behavioral problems, and the related effects are inextricably related to mental health [21]. For example, in adolescents, the chain reaction triggered by changes in clock genes may be involved in biorhythm abnormalities and suicide attempts due to impaired regulation of circadian rhythms and emotional states, with effects on neurodevelopment. Likewise, early trauma and stressful life events can alter the expression of clock genes and lead to suicide attempts. In addition, changes in clock genes may lead to dyssynchrony and abnormality of circadian rhythms, thus damaging both the synchronization of external and internal rhythms and the individuals’ ability to adapt to internal and external environments [22]. This leads to the development of mental health issues and increases the risk of suicide attempts [22]. Another study demonstrated that circadian clock genes can be considered candidates for a molecular link—that is, the association between eveningness and adverse affective functions [23]. Three mechanisms have been proposed to explain the presence of gene-environment correlations (rG×E): passive, active, and evocative models. Passive rG×E describes the relationship between the genotype inherited by the child and the nurturing environment created by the parents. Evocative rG×E refers to a person’s genetically influenced behavior that triggers certain responses in others. Active rG×E describes the link between an individual’s gene-influenced traits and their chosen environment. Thus, genetic influence on the environment could explain part of the association between polygenic scores on mental traits and sleep habits [24].

Genetic comparisons of case and control groups of diseases and the discovery of thousands of genetic loci associated with complex disease risk are important steps in genome-wide association studies (GWAS); This also suggests that genomic information is a potential candidate for improving disease risk assessment, especially when combined with environmental factors [25]. In addition, most G×E studies rely mostly on a single candidate gene, an approach that is moving toward a growing consensus that complex diseases have important polygenic components in which genetic influences are manifestations of the combined additive effects of many variants. Polygenic risk score (PRS), also known as polygenic score (PS), is a score based on variation and/or correlation weight of multiple gene loci. It can be used as the best predictor of phenotypic traits when multiple genetic variations are considered [26, 27]. PRS for candidate genes is a common method to investigate the combined effects of multiple loci and has been widely recognized by researchers. In addition, the use of aggregated statistics published by large GWAS could serve as a promising approach emerging in the pantypic genomics literature, a genome-wide polygenic score (GPS) used to calculate a range of major diseases and clinically relevant traits. These scores can also be used to predict some underlying mental health and physical health phenotypes. Recent studies have shown that PRS for schizophrenia can predict disorders such as nicotine use, anxiety, depression, trauma, and psychological disorders, while neurotic PRS can also predict phenotypes such as anxiety, depression, fear, panic, and neurosis [28]. It also provides a theoretical basis for this study to construct PRS that meet the requirements through the known gene database. Since multiple clock genes are located at important sites across the genome, GWAS focusing on chronotypes have confirmed the role of clock genes [29]. Other studies also demonstrated that circadian clock genes can be considered candidates for a molecular link between chronotype and mental behavioral symptoms [23, 30, 31]. Some studies observed the association with mood and behavior by constructing CLOCK-PRS [32, 33]. Other studies also found that CLOCK genes may be candidate genes for psychological behavior problems [34, 35].

Although GPS is a more comprehensive analysis method, it has stricter requirements on sample size and statistics. In contrast, the use of candidate gene PRS is a concise way to ensure statistical validity and provides a theoretical basis for the construction of the required clock candidate PRS from the known GWAS database in this study [36]. Previous studies on other candidate genes have also been reported; for example, Priess-Grobe and Hyde (2013) showed that MAOA gene could regulate the interaction between 5-HTTLPR gene and negative sexual events and showed gender differences. In girls carrying the low activity allele of MAOA, the level of depression in carriers of 5-HTTLPR S-allele increased significantly after stressful life events, while the interaction between 5-HTTLPR L-allele and stressful life events (SLEs) affected depression in males and only in individuals carrying low-activity MAOA genotype [37]. The results showed that the relationship between gene × gender × environment was related to psychological behavior problems. Qiu et al. found that arginine vasopressin receptor 1-B (AVPR1B) gene has a significant interaction with impulsivity and life events. The rs28373064 polymorphism of AVPR1B gene is associated with suicide attempt in patients with depression. The AA genotype carriers at this locus are more likely to commit suicide under the influence of life events and impulsive traits [38]. Roy et al. assessed the impact of corticotropin-releasing hormone receptor 1 (CRHR1) rs17689918 and its reaction with the family environment on mood and behavioral characteristics, finding various mental health impacts including anxiety, depression, aggression, and antisocial behavior, and identified the influence of gene × environment × gender interaction on the above results [39].

These findings suggest that HRBs may be premised on the inclusion of another regulatory mechanism, including demographic characteristics and impulsivity. Mental health, as one of the common effects leading to HRB, have also been reported in several studies [40, 41], with some studies finding the interaction between clock genes and mental health to be related to sleep behavior problems [42]. Homologous genes involved in animal activity-rest cycles have been described in humans. An individual’s tendency to get up and go to bed more or less early has been observed to be associated with polymorphisms in the *circadian locomotor output cycles kaput*(*clock*) gene for the circadian output cycle [43]. External factors regulate the rhythm, known as synchronizers. Genetically related endogenous factors guide the internal clock, which is responsible for internal time synchronization to coordinate the circadian changes of relevant biochemical, physiological and behavioral parameters within the body [44]. Circadian rhythm is affected by genes and has a certain genetic basis [45]. The genetic background mechanism of the core biological clock has also been preliminarily established [6, 46, 47]. Clock genes are important candidate gene groups for the study of circadian rhythm system and biological clock background. Studies have found that clock genes play an important role in regulating energy homeostasis, and single nucleotide polymorphisms (SNPs) of some clock genes (CRY1, PER1) are also associated with metabolic disorders, obesity and eating habits [48, 49]. In addition, those genes that regulate the biological mechanisms of physiological changes associated with chronotypes are called “clock” or circadian rhythm genes [32].

### Current study

The existing research focuses on a single gene. Although such research can improve our understanding of the specific risk theory model of biological pathways, the results are inconsistent due to the environment interaction effect (which may reflect the study of differences between overall genetic effects and specific genes in a complex polygenic system). Accordingly, the field of genetics has moved toward creating PRS across many genes and showing predictive power in cases where a single gene cannot be detected [International Schizophrenia Consortium et al., 2009]. In addition, most PRS studies are still carried out under a single environmental factor, so genetic analysis in populations with different environments and cultures is needed to improve the universality of PRS predictions. Likewise, the interaction between environmental factors × CLOCK-PRS × mental health and adolescent HRBs has not been investigated. Accordingly, in order to take into account the multiplicity of adolescents’ living environments, this study used CLOCK-PRS to explore environmental influences on adolescent HRBs and consider whether those environmental factors had any regulatory impact on psychological or pathological symptoms. In addition, the study attempts to elucidate the pathophysiological mechanism of adolescent clustering of HRBs from a broader perspective.

## Methods

### Research objects

This study adopts a cross-sectional study design and is based on a nationwide sample survey. These cross-provincial surveys collected data on school-aged children’s health, well-being, and social context every two years in three to four cites in China. From October to November 2021, questionnaires and oral pharyngeal swabs were collected from four middle and high schools in Xuzhou city, Jiangsu province using the cluster sampling method. All students in three classes of each grade in each school were selected for the questionnaire survey. A total of 320 questionnaires were sent out. After reviewing the questionnaire responses for validity and eliminating those that did not meet the requirements of a valid response, 264 questionnaires and their respective respondent were accepted for analysis. The researchers obtained the informed consent of the respondents and their guardians, and the study was reviewed by the Ethics Committee of Anhui Medical University. The power effect was 0.79909. A total of 164 boys (62.1%) and 100 girls (37.9%) with an average age of 14.20 ± 1.88 years were included in these surveys. The researchers note that some scholars have suggested that studies on small samples can also reflect some phenotypic symptoms [36, 50].

### Research methods and experimental content

#### Experimental research methods of oral pharyngeal swabs

Oral samples were obtained by rubbing oral mucosa with a set of sterile DNA-free oral pharyngeal swabs [51].

#### DNA extraction and gel electrophoresis

**DNA extraction**

In this study, the whole blood genomic DNA rapid extraction kit provided by Beijing Beitech Biotechnology Co., Ltd. was used to prepare peripheral blood DNA.

**DNA gel electrophoresis**

After the sample DNA was extracted, the extracted sample DNA was screened using gel electrophoresis. Other detailed were shown in supplement methods.

#### Sequencing method

The BGI-low Pass process is a process based on low-depth whole-genome sequencing (0.1×, 10×) for Genotype Imputation. The reference version used in this study is HG38_NOalt. In their study, Liu et al. replicated 43 of the 58 causal effects using low-depth whole-genome sequencing data from 1,430 individuals in the study [52]. Other studies have also adopted this method [53]. Before the formal experiment of this study, two people were selected to obtain genome sequencing data of different depths by using the resequencing technology. Results of 2×, 5×, and 10× were compared, and the filling accuracy of low-depth resequencing data was evaluated by combining the whole gene sequencing data of 10×.

### Quality control requirements

1. The determination criteria of the PCR product concentration of the DNBSEQ WGS library were as follows: the concentration was greater than 5 ng/µL, and the outbound volume was 30 µL. That is, the total volume of DNBSEQ WGS library was more than 150 ng.
2. The determination criteria for the PCR product fragments of the DNBSEQ WGS library were as follows: the library fragment size was sampled by 2100/CALIper. The library fragment size was 250–450 bp, and the primer dimer contamination ratio was less than 3%.
3. Once the qualified PCR products were detected, the library was cyclized, and the cyclization library was detected using a Qubit fluorescence quantitative analyzer or BMG microplate analyzer.
4. The next step of make DNB can be carried out after the cyclated products of DNBSEQ WGS library pass the detection.

### Research variables

#### Knowledge domain mapping map

In this study, VOS-viewer software (http://www.vosviewer.com/) was used to conduct text recognition and cluster analysis of relevant literature on HRB and risk factors. The resulting data were exported, and related risk factors were sorted out to initially explore the classification methods of HRB risk factors [54]. VOS-viewer is a software tool for processing, visualizing, and mining associations (including modularity, clustering, and association strength) within web data in the form of knowledge domain mapping maps. VOS-viewer can be used to build networks of scientific publications, scientific journals, keywords, or terms. Data types include Network Data, Bibliographical Data, and Text Data. Data import formats include file formats from databases such as Web of Science, Scopus, Dimensions, Lens, PubMed, RIS, or Crossref JSON. VOS-viewer’s results provide three forms of Visualization: Network Visualization, Overlay Visualization, and Density Visualization (see Figure S1).

#### Questionnaire survey

Mental health

1. The Psychological Sub-Health Scale using the adolescent mental health assessment questionnaire of concise evaluation of the object of study in the past two weeks including emotional problems, conduct disorder and social adaptation of psychological sub-health status [55], questionnaire items including 15 questions, answer options were “① for 3 months or more,” “② for more than 2 months,” “③ for more than a month,” “④ Lasts more than 2 weeks,” “⑤ lasts more than 1 week,” “⑥ No or less than 1 week.” In this study, the Cronbach α coefficient of psychological sub-health was 0.949.
2. A Patient Health Questionnaire (PHQ-9) was used to evaluate depression in the subjects [56]. Four answer options were included in the questionnaire: “① not at all;” “② several days;” “③ more than half of the days;” and “④ almost every day.” In this study, the Cronbach α coefficient of the PHQ was 0.938.
3. The Generalized Anxiety Scale (GAD-7) was used to evaluate anxiety of the subjects [57]. The questionnaire had seven items, and the answer options were “① not at all,” “② several days,” “③ more than half of the days,” and “④ almost every day.” In this study, the Cronbach α coefficient of the generalized anxiety scale was 0.944.

**Health risk behaviors**

HRBs were measured using the Youth Risk Behavior Surveillance System (YRBSS) [58–60]. We extracted 14 HRBs: smoking, drinking, skipping breakfast, excessive weekday and weekend screen time (ST), physical inactivity, fast food/takeaway and sugar sweetened beverages (SSBs) consumption, eat less vegetables and fruits, suicide ideation (SI), suicide plan (SP), suicide attempt (SA), non-suicidal self-injury (NSSI). Additional details about the data gathered can be found in the supplemental text.

**Chronotype**

The chronotypes were measured using the MEQ (Morningness-Eveningness Questionnaire). The morning/evening preference was categorized as morning (“definitely a morning person”), intermediate (“between a morning person to an evening person”) and evening (“definitely an evening person”) [61]. The suggested demarcations were 4–7 for definite eveningness, 8–11 for medium eveningness, 12–17 for intermediate type, 18–21 for medium morningness, 22–25 for definite morningness. The Cronbach’s α coefficient for the MEQ was 0.628.

### Data analysis

#### Introduction to research methods of PRS

The effect of a single SNP locus on multifactorial diseases is often weak. Therefore, it is difficult to fully reflect the true association between gene variation and outcome variables in a single mutation or single gene study. Instead, the polygenic risk score (PRS) method, an important method in the study of polygenic diseases, can better reflect the association between overall gene variation and outcome variables [62], especially when the gene score contains many common variations with small effects [63]. Even when a single genetic variation has little or no effect, PRS can explain a certain proportion of the variation associated with risk factors and diseases, which makes PRS a very popular method for genetic association research [63].

#### Calculation of PRS

PRS can be divided into unweighted and weighted PRS. Unweighted PRS is obtained by adding up the number of mutant alleles in the loci that construct PRS [62]. Weighted PRS is obtained by the product of the number of mutant alleles and the corresponding weight of the gene loci constructing PRS [64, 65]. The weight was calculated according to the genetic effect of each allele on the outcome variable, while the genetic effect of each allele on the outcome variable was calculated by logistic regression with the outcome variable as the dependent variable and each SNP as the independent variable [66].

The specific calculation formula is as follows: unweighted PRS = SNP_l_ + SNP_2_ +… + SNP_i_, weighted PRS = (W_1_ × SNP_l_ + W_2_ × SNP_2_ +… + W_i_ × SNP_i_)/(W_1_ + W_2_ +… + W_i_), where i is the number of SNP loci involved in PRS construction, the value of SNP_i_ depends on the number of mutant alleles in gene loci—represented by 0, 1 and 2, respectively, and W_i_ is the weight of each gene loci. Unweighted PRS was used in this study.

#### Interaction analysis

In multivariate statistical analysis, interaction refers to the effect of a specific factor on the outcome variable that is affected by other factors. In other words, the interaction exists when the effect of two factors on the outcome variable is not equal to the combined effect of the two factors alone [63, 65].

#### Descriptive statistical analysis

Questionnaire data entry and association analysis were the same as study 2. Counting data were represented by percentile (%), and measurement data were represented by mean plus or minus standard deviation (*x ± s*). An independent sample *t*-test was used between the two groups, and one-way analysis of variance (ANOVA) was used for multi-group analysis to compare scores of chronotype of adolescents with different demographic characteristics. The Chi-squared test was used to analyze the distribution of clustering of HRBs in adolescents with different population characteristics.

### Statistical analysis

The methods used to describe continuous variables in this study are mean and standard deviation (SD), while categorical variables are described in terms of frequency and percentage. Chi-square test was also used for categorical variables, and univariate ANOVA was used to assess differences for continuous variables. To identify clusters of HRBs, latent class analysis (LCA) was performed using Mplus 7.4 to identify homogeneous, mutually exclusive patterns of 15 HRBs. Multivariable logistic regressions were conducted to evaluate the relationships between social ecological, chronotype, mental health, and HRBs, presented as adjusted odds ratios (aORs) with 95% confidence intervals (CIs). In the multivariable logistic regressions, adjustment was made for gender, age, regional areas, school, urban/rurality, parents’ education level, economic status of family, only child, and resident student. All analyses were conducted using SPSS software, Windows version 23.0.

### Sensitivity analysis

Sensitivity analysis was also carried out in this study, including the following: Model 1 crude model without controlling covariates; Model 2 controlled for gender and age; Model 3 controls, on the basis of Model 2, parental education level, family residence (rural, township, and city), whether the family is the only child, family economic status, number of friends, and academic performance. Logistic regression analysis was performed for chronotype with clustering of HRB. To explore the correlation between chronotype and co-occurrence of HRB, and calculate the co-occurrence of HRB, the study used the following methods. With respect to physical activity, smoking, drinking, weekday and weekend ST, takeaways/fast food, SSBs, breakfast, vegetables and fruits, NSSI and SI, suicide planning and suicide attempts of 14 kinds of actions carried out in accordance with the presence of risk classification, divided into two, according to standards according to the variable will occur for each HRB danger, for 1 min, the number of all HRB in each research object was added up to form a “co-occurrence index” [67], which divided the HRB co-occurrence into 0, 1–3, and 4 or more types.

## Results

### Identification of key factors of HRBs based on multi-source data

HRB is the result of a combination of multiple factors and is closely related to a variety of risk factors. The risk factors affecting HRB were comprehensively analyzed in accordance with existing databases and literatures. PubMed database was searched, and the retrieval strategy was constructed with “adolescent health risk behavior **AND** risk factors” as the keyword. The search generated 29,140 articles related to HRB risk factors. The article information was imported into VOS-viewer software, and the knowledge domain mapping map was drawn by analyzing the bibliographic fields of the articles (see Figure S1).

According to the clustering results of HRB risk factor research cataloging information, Figure S1(A) demonstrates the clustering of information fields, showing four prominent areas: the red areas focus mainly on “students,” “violence,” “suicidal behavior,” “adolescent risk behavior,” “health problems,” “high school student,” and “gene;” the yellow area centers mainly on “behavioral risk factor surveil,” “binge drinking,” “behavior risk factor,” and “chronic disease;” the blue area revolves mainly around “drug use,” “sexual behavior,” “high risk behavior;” and the green area is dominated by “cardiovascular disease,” “mortality,” and “physical activity.” Figure S1(B) shows the visualization result of project density, whose project distribution is consistent with network visualization. Each node in the project density visualization has a color that indicates the density of the project on that node. The colors range from blue to green to red. The more items near a node, the higher the weight of adjacent items, the redder the color of the node. Conversely, the fewer the number of items near a node, the lower the weight of adjacent items, the closer the node is to blue. Figure S1(C) shows the same item for overlay visualization as for web visualization, the difference being the color of the item. The colors of the projects range from blue to green to red, from far to near, corresponding to the year of the project cluster. According to the trend of node color changes in the figure, researchers’ orientation shifted from sexual behaviors and communicable diseases to suicidal, NSSI, sedentary behaviors, psychological problems, etc.

### General demographic statistics

Among the 264 students included in the analysis, 164 were male and 100 were female. The median student age was 14.20 ± 1.88 years; 16 (6.0%) students lived in rural areas, 42 (15.8%) lived in towns, and 206 (78.4%) lived in cities. Of the 264 students, 69 were only children (26.0%) and 195 non-only children (74.0%); 95 (36.2%) had junior high school education or below, and 169 (63.8%) had senior high school education or above; 118 (44.5%) of mothers had junior middle school education or below, and 146 (55.5%) had senior high school education or above; among them, 24 (9.1%) were poor, 181 (68.7%) were moderate, and 59 (22.3%) were good. Fifty-eight (21.9%) had fewer than two friends, 87 (32.8%) had three to five friends, and 119 (45.3%) had more than six friends. Eighteen students (6.8%) had a light study load, 158 (59.6%) had a medium study load, and 89 (33.6%) had a heavy study load. One hundred-thirty in grade one and 135 in grade one. The results between general demographic and chronotype are shown in Table 1.

**Table 1:** The prevalence of demographic characteristics among chronotype

Variables	Total N(%)	Eveningness n(%)	Intermediate n(%)	Morningness n(%)	*χ*^2^ /F value
Age	264	14.85 ± 1.52	14.21 ± 2.01	13.91 ± 1.70	3.01*
Gender					6.92*
Male	157(62.1)	14(8.9)	91(58.0)	52(33.1)	
Female	103(37.9)	20(19.4)	58(56.3)	25(24.3)	
Residential areas					2.48
Country	16(6.1)	2(12.5)	7(43.8)	7(43.8)	
Town	42(15.8)	5(11.9)	27(64.3)	10(23.8)	
Urban	204(78.1)	27(13.2)	117(57.4)	60(29.4)	
Only child					0.16
Yes	69(26.1)	9(13.0)	41(59.4)	19(27.5)	
No	193(73.9)	25(13.0)	110(57.0)	58(30.1)	
Father’s education					2.77
Junior high and below	95(36.0)	8(8.4)	58(61.1)	29(30.5)	
High school or technical secondary school	95(36.4)	15(15.8)	53(55.8)	27(28.4)	
Junior college or above	72(27.7)	11(15.3)	40(55.6)	21(29.2)	
Mother’s education					5.62
Junior high and below	117(44.7)	13(11.1)	76(65.0)	28(23.9)	
High school or technical secondary school	87(32.6)	12(13.8)	43(49.4)	32(36.8)	
Junior college or above	58(22.7)	9(15.5)	32(55.2)	17(29.3)	
Family economic conditions					9.24
Very bad	5(1.9)	2(40.0)	2(40.0)	1(20.0)	
Worse	19(7.2)	2(10.5)	10(52.6)	7(36.8)	
Medium	180(68.6)	22(12.2)	112(62.2)	46(25.6)	
Better	43(16.7)	6(14.0)	21(48.8)	16(37.2)	
Very good	15(5.7)	2(13.3)	6(40.0)	7(46.7)	
Friends number					3.66
No	9(3.4)	0(0.0)	5(55.6)	4(44.4)	
1–2	49(18.6)	6(12.2)	27(55.1)	16(32.7)	
3–5	85(32.6)	9(10.6)	51(60.0)	25(29.4)	
6 or more	119(45.5)	19(16.0)	68(57.1)	32(26.9)	
Learning burden					5.72
Light	18(6.8)	0(0.0)	10(55.6)	8(44.4)	
Medium	155(59.5)	22(14.2)	85(54.8)	48(31.0)	
Heavy	89(33.6)	12(13.5)	56(62.9)	21(23.6)	

### Clustering and distribution of adolescent health risk behaviors

The HRBs in this study included 14 types, including lack of physical inactivity, smoking, drinking, ST on weekdays and workdays, takeaways/fast food, SSB, skipping breakfast, insufficient vegetable intake, insufficient fruit intake, NSSI, suicidal ideation, planning, and attempt. According to the latent category results of HRBs, the two types of models were selected based on lower BIC and ssaBIC and higher entropy (0.872), and the mean posterior category membership probability scores (0.749–0.898) between groups were acceptable. Figure S2 shows the project response probabilities of the two classes of HRBs models and the 14 HRBs of each class. Category 1 was characterized by a high probability of exposure to 14 HRBs; therefore, we labeled this potential category as “high HRBs” (17.2%) and the low individual composition in category 2 as “low HRBs” (81.8%). See Table S1 and Figure S2 for the results.

### Clustering of adolescent HRBs

#### General demographic characteristics of adolescents

Among the 264 adolescents, the median age of the high-level group was 13.93 ± 1.66 years and that of the low-level group was 14.26 ± 1.92 years. No statistical significance in gender, residential areas, only child, educational level of parents, family economic status, number of friends, and learning burden among the two groups. See Table S2 for the results.

#### Association between mental health and adolescent clustering of HRBs

In this part of the investigation, the Psychological Sub-Health Scale, Patient Health Questionnaire Depression Self-Rating Scale (to evaluate depression) and Generalized Anxiety Scale (to evaluate anxiety) were used to evaluate the level of adolescents’ mental health. The scores of the three scales in the high-level group were all higher than those in the low-level group, and the differences were statistically significant (see Table S3 and S4).

### Association between chronotype and adolescent psychological and behavioral problems

#### Univariate and multivariate analysis of chronotype and adolescents’ psychological and behavioral problems

A logistic regression model was used to investigate the influence of chronotype on adolescent clustering of HRBs and mental health. Clustering of HRBs (low-level group = 1, high-level group = 2), HRB co-occurrence (4 or less = 1, 5 or more = 2), psychological sub-health (low = 1, high = 2), depression (no = 1, yes = 2) and anxiety (no = 1, yes = 2) were used as dependent variables, and chronotype were used as independent variables (low-risk = 1, medium-risk = 2, high-risk = 3, low-risk as control group). The results of univariate logistic regression analysis showed that the eveningness was associated with the increased risk clustering of HRBs, high-level psychological sub-health, high-level depression and anxiety among adolescents, and the difference was statistically significant.

Adjustment of gender, age, parents’ educational level, family residence, only-child status, family economic conditions, self-assessment, number of friends, and learning burden after variables, the analysis results show that the later chronotype with the same high-level health risk adolescents’ clustering of HRBs had high-level psychological sub-health and an increased risk of depression and anxiety. Moreover, the difference was statistically significant. The results are shown in Table 2 and Table S5 to S8.

**Table 2:** Association between chronotype and clustering of HRBs in adolescents

Variables	Total model			Male		Female	
	Low	High		Low	High	Low	High
Chronotype							
Eveningness	1.0	2.18(0.78,6.09)		1.0	3.84(0.91,16.17)	1.0	1.03(0.21,5.05)
Intermidiate	1.0	1.13(0.52,2.42)		1.0	1.18(0.43,3.27)	1.0	0.90(0.24,3.36)
Morningness	1.0	1.0		1.0	1.0	1.0	1.0

Total model were adjusted for gender, age, educational level of parents, family residence, only child, self-rated family financial status, number of friends and learning burden

#### Chronotype and mediate moderation analysis of adolescents’ psychological and behavioral problems

The process model was used to investigate the influence of chronotypes, gender, and mental health on adolescent clustering of HRBs, with HRB co-occurrence as the dependent variable, chronotypes as the independent variable, gender as the moderating variable, and mental health as the mediating variable. To analyze the association between chronotypes and clustering of HRB and the moderating effects of gender and the mediating effects of mental health.

After adjusting for age, parental education level, family residence, only child status, self-rated family economic status, number of friends, and learning burden, the results of process adjustment analysis showed that eveningness and gender were not associated with anxiety symptoms but correlated with increased risk of HRB co-occurrence. Moreover, a statistically significant difference was observed between gender and chronotype among HRB co-occurrence (β = 0.0003, 95% CI: 0.0001, 0.001), while no significant difference was observed in clustering of HRBs (Table S9–Table S12).

### Association of genetic factors with risk of HRBs

#### Genome-wide association analysis of clustering of HRBs

A GWAS was conducted in a small sample of adolescents to find the genetic variation loci related to clustering of HRBs and further analyze and evaluate the heritability and genetic structure of adolescent clustering of HRBs according to the results of GWAS analysis. After genotypic data and sample quality control, a total of 258 participants and 3 807 619 SNPs were included in the analysis. R software was used to conduct linear regression analysis for each SNP and clustering of HRBs in additive mode. The Manhattan map based on GWAS summary statistics (Figure S3) shows that 372 SNPs (n = 372) exceed the genome-wide significance level (P < 5 × 10^− 4^). The Q-Q plot of clustering of HRBs GWAS (Figure S4) shows the correlation between SNPs and clustering of HRBs, and the genome inflation factor λ = 1.04.

#### Genome-wide association analysis of HRB co-occurrence

R software was used to conduct linear regression analysis for each SNP and HRB co-occurrence in additive mode. The Manhattan map based on GWAS summary statistics (Figure S5) shows that 543 SNPs (n = 543) exceed the genome-wide significance level (P < 5 × 10^− 4^). The Q-Q plot of HRB co-occurrence GWAS (Figure S6) shows the correlation between SNPs and HRB co-occurrence, and the genome inflation factor λ = 1.3.

SNPs with significant levels were screened and five independent sites of health risk behavior were identified: adherens junction-associated protein-1 (AJAP1), ATP synthase subunit S-like protein (ATP5SL), DISC1FP1, low density lipoprotein receptor-related protein 1B (LRP1B), and prokineticins receptor 2 (PROK2).

### Genetic distribution of adolescent clustering of HRBs

#### Basic information of candidate gene SNPs

The screening of candidate genes in this study was divided into three steps. First, according to the results of GWAS and the method of P value < 5 × 10^− 8^, among the results of clustering of HRBs and HRB co-occurrence, only part of the SNP sites of PROK2 gene met the requirements. Second, 372 genes were screened from clustering of HRBs according to P value < 5 × 10^− 4^. Thirdly, NPAS2 (rs13025524, rs3768984, and rs11673746) and ARNTL (rs2278749) meet the requirements of P < 0.05. According to previous references, in the final candidate gene analysis, 49 SNPs were included in 9 candidate genes including CLOCK, ARNTL, NPAS2, PER1, PER2, PER3, CRY1, CRY2, and NR1D1, and the SNPs information of each gene is shown in Table S13. The results also show the differences between TREND and ALLELIC models for alleles as well as the dominant model (Dom) and recessive model (Rec).

#### Association between CLOCK-PRS and adolescent clustering of HRBs

**Association between CLOCK-PRS and adolescent clustering of HRBs**

Table 3 describes the association between CLOCK-PRS and adolescent clustering of HRBs. The prevalence of adolescent clustering of HRBs with high genetic risk was higher than that with low OR medium genetic risk, but the difference was not statistically significant (P > 0.05). Logistic regression results showed that high genetic risk was associated with high level clustering of HRBs, OR = 1.40, 95% CI = 0.66, 2.97; however, the difference was not statistically significant, and no statistical significance was found after adjusting covariates (Table 3, P > 0.05).

**Table 3:** Association between CLOCK-PRS and clustering of HRBs in adolescent

PRS	Total N(%)	High n(%)	Low n(%)	*OR*(95%*CI*)^a^	*OR*(95%*CI*)^b^
High	93(36.0)	21(44.7)	72(34.4)	1.40(0.66,2.97)	1.26(0.57,2.76)
Medium	69(26.7)	9(26.2)	60(27.8)	0.94(0.42,2.13)	0.89(0.39,2.04)
Low	96(37.3)	17(36.2)	79(37.8)	1.0	1.0

a: crude model; b: Adjusted for gender, age, educational level of parents, family residence, only child, self-rated family financial status, number of friends and learning burden

**Subgroup analysis based on demographic characteristics**

This study also to CLOCK-PRS with clustering of HRBs on different demographic characteristics, including gender, age, parents’ educational level, residential areas, only-child status, self-reported family economic conditions, number of friends, and learning burden, the results showed that the only-child status influence on adolescents clustering of HRBs can adjust CLOCK-PRS (Table S14, P < 0.05); no significant differences were observed in other demographic characteristics (P > 0.05).

### Impact of interaction between CLOCK-PRS and chronotypes on adolescent HRBs

#### Association between the interaction of CLOCK-PRS and chronotype and the adolescent clustering of HRBs

The study used the logistic regression model to investigate the genetic interaction and the chronotypes influence on clustering of HRBs. To analyze associations between chronotypes and genetic risk interactions, and HRB clustering in adolescence, clustering of HRBs (1 = low-level group, high-level group = 2) was the dependent variable, and the chronotype (eveningness = 1, intermediate = 2, morningness = 3) and CLOCK-PRS (low genetic risk = 1, medium genetic risk = 2, low genetic risk = 3) were the independent variables. Univariate logistic regression analysis showed that the interaction between eveningness and high genetic risk was associated with an increased risk clustering of HRBs in adolescents.

After adjusting for gender, age, parents’ educational level, family residence, only-child status, family economic conditions, self-assessment, numbers of friends, and learning burden, the analysis shows that adolescents evening chronotypes and genetic risk for interaction is also associated with the increase clustering of HRBs, and the difference was statistically significant (Table 4, P < 0.05).

**Table 4:** Association of CLOCK-PRS and chronotype interactions with clustering of HRB in adolescents

Variables	Model 1			Model 2		Model 3	
	Low	High		Low	High	Low	High
PRS + chronotype							
High PRS＋eveningness	1.0	3.50(0.56,22.03)		1.0	3.72(0.58,23.70)	1.0	4.02(0.61,26.51)
High PRS＋intermidiate	1.0	1.84(0.46,7.44)		1.0	1.75(0.43,7.11)	1.0	1.69(0.40,7.04)
High PRS＋morningness	1.0	1.52(0.32,7.16)		1.0	1.44(0.30,6.80)	1.0	1.69(0.34,8.35)
Medium PRS＋eveningness	1.0			1.0		1.0	
Medium PRS＋intermidiate	1.0	1.62(0.39,6.62)		1.0	1.66(0.40,6.82)	1.0	1.72(0.40,7.35)
Medium PRS＋morningness	1.0	1.47(0.29,7.45)		1.0	1.51(0.30,7.69)	1.0	1.65(0.31,8.77)
Low PRS＋eveningness	1.0	5.25(1.05,26.20)*		1.0	5.57(1.10,28.20)*	1.0	5.66(1.06,30.22)*
Low PRS＋intermidiate	1.0	0.93(0.21,4.10)		1.0	1.06(0.24,4.70)	1.0	1.12(0.25,5.15)
Low PRS＋morningness	1.0	1.0		1.0	1.0	1.0	1.0

Model 1 : crude model ; Model 2: Adjusted for gender, age; Model 2: Adjusted for gender, age, educational level of parents, family residence, only child, self-rated family financial status, number of friends and learning burden

#### CLOCK-PRS and chronotype interaction HRB co-occurrence and mental health

Univariate logistic regression analysis showed that the interaction between high genetic risk and high chronotype was associated with increased risk of adolescent depression (OR = 2.63, 95% CI = 0.92, 7.49); however, no significant difference was observed (Table S15, P > 0.05).

After adjusting the variables such as gender, age, parental education level, family residence, only-child status, self-rated family economic status, number of friends, and study burden, the results of multiple logistic regression analysis P < 0.05 showed that the interaction of eveningness and high CLOCK-PRS risk was also associated with increased anxiety among adolescents (OR = 4.01, 95% CI = 1.29, 12.50), and the difference was statistically significant (Table S16, P < 0.05).

#### Moderation analysis of chronotypes and CLOCK-PRS and adolescents’ psychological and behavioral problems

The process model was used to investigate the effects of chronotypes, CLOCK-PRS, and mental health on adolescent clustering of HRBs, with HRB co-occurrence as the dependent variable, chronotypes as the independent variable, CLOCK-PRS as the moderating variables, and mental health as the mediating variables. Accordingly, this model analyzed the association between chronotypes and clustering of HRBs and the moderating effects of CLOCK-PRS and the mediating effects of mental health.

After adjusting for gender, age, parental education level, family residence, only-child status, self-rated family economic status, number of friends, and learning burden, the results of process adjustment analysis did not reveal a interaction between chronotypes and CLOCK-PRS and anxiety symptoms, and the difference was not statistically significant. The results are shown in Tables S17 and S18. No interactions were observed between chronotypes and CLOCK-PRS and psychological sub-health and depression (Tables S19 and S20).

## Discussion

### Principal findings

In accordance with the adolescent behavioral health and monitoring research and based on the premise of school, the relevant physical examination and investigation were conducted among the adolescent samples. The results of this study investigated the relationship between chronotype, clock-PRS and their mental health and HRBs. Chronotypes in circadian rhythms are thought to be closely associated with behavioral characteristics associated with depression, and in a theoretical derivation model of the effect of chronotypes on HRBs, multiple measures of hrb were examined using the LCA approach. First, in our study, the high-prevalence cluster of HRBs was 17.2%. Secondly, interaction effect was observed between chronotype and CLOCK-PRS on HRBs and mental health; we examined the mediating effect of mental health on the relationship between HRBs and chronotype, as well as the moderating role that CLOCK-PRS played in the relationship. Thirdly, our results provided strong support for study hypotheses regarding different factors and potential mechanism of adolescent HRBs. Finally, our results provide strong support that chronotypes report a greater risk propensity for mental health and behavioral problems, supporting previous findings showing an association between chronotypes and mental health and HRBs. In addition, the results of this study also explored the possible correlation between chronotype and CLOCK-PRS interaction on adolescent psychological behavior problems by constructing PRS.

### Compared with other studies

Nocturnal habits in people are associated with poorer mental health symptoms, consistent with previous studies [68–70]. This is especially the case with anxiety symptoms in women, suggesting that nocturnal chronotype may be a risk factor for adverse emotional behavior problems. Previous hypotheses explain the pathophysiology of how circadian rhythm disturbances contribute to the development of depression. One idea is that circadian dysregulation alters circadian rhythms at the neuroendocrine or cellular molecular level, making this part of the population more susceptible to depression. In addition, genetic vulnerabilities, such as mutated clock genes, can lead to changes in the processes that produce and carry or synchronize with circadian rhythms, which further leads to phase changes in the sleep-wake cycle [71], disrupting the rhythmic activity of neurotransmitter systems involved in mood regulation, including alterations in serotonin and dopamine. This may lead to a greater likelihood of nocturnal preference and the subsequent development of depressive symptoms [10]. While we did not find a correlation between CLOCK-PRS and HRBs in our study, there are some studies that explain why this part of the results could not be fully replicated, possibly due to false-negative results, false positive results, or true heterogeneity between studies. In psychiatric research, false-negative results are usually caused by a lack of capacity, either due to a small sample size or poor quality of phenotype or genotyping. This study’s results could possibly be attributed to insufficient sample size.

Moreover, unavoidable objective factors, like demographic factors (gender and age), modulate the morning-to-evening characteristics, consistent with our findings. Age and sex can explain much of the variation in chronotype; however, genetic variation itself is also an important factor [72]. In addition, we explored the moderating role of gender. The literature investigating potential mediators of chronotype-HRB relationships shows mixed results regarding mental health as a mediator. Our intermediate-moderate model shows that eveningness is associated with higher mental health and higher HRB, which are both modulated by sex. Although sex differences in circadian rhythm types have been demonstrated before [72], a few reasons could underline why there is no regulatory effect on sex. The size of the effects reported in Landler’s paper and in our study are small in number, therefore, it is not surprising that their results may not have been observed.

Moreover, we explored the association between chronotype preferences and HRB patterns; in our results, we found that eveningness was correlated with HRB co-occurrence but not correlated with clustering of HRB. The possible mechanism for the correlation was that daily circadian rhythms influence variations in human physiology and behavior [10]. Other studies have quantified the relationship between the evening chronotype and mental health [10]. Circadian typologies are currently understood to be a behavioral trait related to depressive disorders. An individual’s chronotype is determined by genetic variations in clock genes [10, 73] and environmental factors [74, 75]. In addition, recent GWAS have identified specific genetic variants associated with self-reported chronotypes [29, 76].

As mentioned earlier, several hypotheses have been proposed to explain how circadian rhythm disturbances, including at the genetic level, contribute to understanding the pathophysiology of mental health and HRB occurrence. Our study further explored the relationship between chronotype and CLOCK-PRS and mental health and behavior problems because the influence of gene level on psychological and behavioral outcomes depends on other factors. This study injected chronotype variable on the basis of focusing on clock genes to further increase the theoretical basis for understanding gene-environment interaction. This also implies that genetic factors may contribute to the association between chronotype and mental health dimensions [70]. A GWAS in a UK Biobank cohort of 100,420 middle-aged adults found that results using gene-set enrichment analysis of loci associated with fear and behavioral defense responses suggest that evening chronotypes may be more prone to mental health disorders and play a role through behavioral inhibition [77]. Taylor and Hasler also concluded that specific genes associated with chronotypes and psychiatric disorders include CLOCK, PER3, ARNTL, and TIM [70, 77, 78]. Moreover, some existing evidence support a link between clock genes and comorbidities of alcohol use and depression [79, 80]. In addition, based on the Taylor and Hassler study [70], which further provides compelling evidence of triadic interaction effects in linking clock genes, stress, and behavioral problems, and proposes potential neurobiological mechanisms [79], sleep habits and genetic and psychological issues play a role in HRBs.

### Limitations and strengths

In our sample, we did not observe a significant correlation between the measured CLOCK-PRS and time type and HRB [32]. Available evidence suggests that chronotypes are highly influenced by different factors, including geographic factors and gender differences [79], which may explain one of the reasons for the lack of correlation in our sample. Although gender differences in circadian rhythm types have been demonstrated before [72], there are some factors that may explain why gender did not have a regulatory effect in our study, namely the objective factor of differences in sample selection. Possible reasons include the small sample size [10] and the size of the candidate gene effect. Although age and gender play a moderating role in chronotype and mental health and behavioral problems, age and gender do not play a moderating role in gene and chronotype and mental health and behavioral problems. The sample size and candidate gene number will be further expanded in the future.

## Implications and future directions and conclusions

The results of this study have certain clinical and practical significance. First, we found that people with poor mental health are more likely to develop HRBs, suggesting that treatments should focus on adolescents with psychological problems because they have a potential role in influencing HRB. Second, evening chronotype, as a factor associated with both mental health and behavioral problems, identifies individuals at risk according to chronotype and mental health for early intervention, which can further deepen and guide our understanding, ultimately improving the prevention, diagnosis, and treatment of mental health and behavioral problems, and addressing their sleep habits and future health risks. Third, while the relationship between the genetic level and mental health in this study may be small, it is not surprising given the complexity of genetic, environmental, and social factors that influence the onset and maintenance of affective disorders. However, these results do indicate that a discussion at the genetic level alone can be meaningful. Accordingly, we plan to further explore the possible reasons for the association between chronotype and psychobehavioral problems in the future.

### Electronic supplementary material

Below is the link to the electronic supplementary material.

**Supplementary Material 1: Table S1.** Screening results of latent categories of health risk behaviors

## Data Availability

The datasets generated for this study are available on request to the corresponding author.

## List of abbreviations

ANOVA: Analysis of variance
CI: Confidence intervals
GPS: Genome-wide polygenic scores
GWAS: Genome-wide association studies
HRB: Health risk behaviors
LCA: Latent class analysis
MEQ: Morningness-Eveningness Questionnaire
NSSI: Non-suicidal self-injury
PHQ: Patient Health Questionnaire
PRS: Polygenic risk score
PS: Polygenic score
SA: Suicide attempt
SD: Standard deviation
SI: Suicide ideation
SLE: Stressful life events
SNP: Single nucleotide polymorphisms
SP: Suicide plan
SSB: Sugar sweetened beverages
ST: Screen time
YRBS: Youth Risk Behavior Survey
YRBSS: Youth Risk Behavior Surveillance System
