# Supplementary material for: Role of polygenic risk scores in the association between chronotype and health risk behaviors
Source: BMC Psychiatry. 2023 Dec 20;23:955. doi: 10.1186/s12888-023-05337-z (PMC10731716; doi:10.1186/s12888-023-05337-z)
Supplement: Supplementary file 1 — Supplementary Material 1: Table S1. Screening results of latent categories of health risk behaviors [file 12888_2023_5337_MOESM1_ESM.docx]

**Methods**

*2.1 Research Objects*

We use data from a collaborative cross-provinces survey of school-aged children’s health and well-being, examined in their social context, conducted every 2 years in 3-4 cites in China. From October to November, 2021, questionnaires and oral pharyngeal swabs were collected from 4 middle and high schools in Xuzhou city, Jiangsu province using cluster sampling method. All students in 3 classes of each grade in each school were selected for questionnaire survey. We sent out 320 questionnaires, checking the quality of the questionnaire, on a regular, contradiction between each response is courtesy (there is a clear logic error) or continuous more than 5 questions did not answer the questionnaire to eliminate, eliminate invalid questionnaire 20, recycling effective questionnaire 300, including questionnaire and sequencing samples to check again, finally select 264 people for analysis.

*2.2 Research methods and experimental content*

(1) Sampling tools: three sets of oral and pharyngeal swabs, including sterile swab collection, preservation tube and plastic bag; label paper (subject's first letter capital + date of birth); drink mineral water.

(2) Sampling method:

① 30 minutes before the sampling, the teenagers were asked to line up, distribute drinking water, and tell them to wash and clean their oral impurities;

② When sampling, wear gloves, hold the mandible in one hand, hold the upper 1/3 part of the cotton swab in the other hand, and put the remaining 2/3 part into the mouth. Wipe the cotton swab from the inside of the buccal mucosa (inside the cheek) up and down for more than 20 to 30 times (rotate the cotton swab from time to time), and then gently remove the cotton swab;

③ Choose the left and right sides of the different parts of the oral wall, with the same method to collect samples, each person should scrape at least three cotton swabs;

④ After the collection, open the preservation tube and put the oral swab into the preservation tube.

(3) Precautions:

① Avoid touching the tip of cotton swab with hands;

② After sampling, gently remove the swab to avoid touching the mouth or other parts of the mouth;

③ After the oral swab test, it should be put in the refrigerator at -20℃ to avoid moldy oral swab affecting the detection;

④ Label the oral swabs of different people (corresponding to the questionnaire).

2.2.4 Quality control requirements

(1) Determination criteria of PCR product concentration of DNBSEQ WGS library: the concentration is greater than 5 ng/μL, and the outbound volume is 30 μL, that is, the total volume of DNBSEQ WGS library is more than 150 ng.

(2) Determination criteria for PCR product fragments of DNBSEQ WGS library: the library fragment size was sampled by 2100/CALIper, the library fragment size was 250 bp-450 bp, and the primer dimer contamination ratio was less than 3%.

(3) Detection of cyclization products of DNBSEQ WGS library. After qualified PCR products were detected, the library was cyclized and the cyclization library was detected by Qubit fluorescence quantitative analyzer or BMG microplate analyzer.

(4) The next step of make DNB can be carried out after the cyclated products of DNBSEQ WGS library pass the detection.

*2.3 Research Variables*

2.3.2 Questionnaire survey

(1) Physical inactivity, the use of "Physical Activity Rating Scale" (PARS-3) questionnaire 3 items, 1) the first is about the intensity of exercise, "in the past month, when you do physical exercise, most of the following intensity of exercise will be selected? The answers include: ① light exercise (such as walking on the playground, doing radio exercises, etc.); ② the movement of small intensity, low intensity (recreational recreational ball games, including the volleyball and table tennis, jogging, tai chi, etc.), ③ moderate intensity, more intense lasting movement (such as cycling and running more than half an hour), ④ shortness of breath, sweating a lot of great strength, but not lasting movement properties of ball games (game), ⑤ rapid breathing, sweating a lot, high intensity of sustained and lasting exercise (such as running, spinning, swimming, etc);

2) The second one is about the evaluation of the duration of exercise, "how many minutes does the activity of the above intensity last?" the answers include ① ≤ 10 minutes, ② 11-20 minutes, ③ 21-30 minutes, ④ 31-59 minutes, ⑤ ≥ 60 minutes;

3) The third is the frequency of physical activity, "how many times have you done these activities?" including ① 1 month ≤ 1 time; ② 2 ~ 3 times a month; ③ 1~2 times a week; ④ three to five times a week; ⑤ about once a day;

Calculate the sports activities of youth, the intensity and frequency of option value assignment for 1 ~ 5 minutes, the time assignment of 0 ~ 4 points, the calculating formula for physical activity the strength * time * frequency, in accordance with the 50 percentile is divided into low activity and high activity, P_50_ or less, for lack of physical activity > P_50_ for sufficient physical activity;

(2) Vegetables and fruits (V&F): "How many servings of vegetables have you usually eaten per day in the last month?" and "How many servings of fruit have you usually eaten per day in the last month?" The answers were "no", "less than 1 portion", "1 portion", "2 portions", "3 portions or more", and "1 portion" and below were defined as insufficient intake of vegetables and fruits, and "2 portions" and above were defined as adequate intake of vegetables and fruits.

(3) Smoking: "How many days in the past month have you smoked at least one cigarette?" The answers included "no", "1 or 2 days", "3 ~ 5 days", "6 ~ 9 days", "10 ~ 19 days", "20 ~ 29 days" and "all 30 days". "no" was defined as no smoking behavior, and "1 day" or more was defined as smoking behavior.

(4) Alcohol consumption: "How many days in the last month have you had at least one glass of wine (a glass of wine is equivalent to half a bottle/can of beer, a small glass of white wine, a glass of wine or yellow rice wine, rather than a sip)"; It also asked "If you're a girl, how many days do you have at least four drinks in an hour or two, and if you're a guy, how many days do you have at least five drinks in an hour or two?" The answers included "no", "1 day", "2 days", "3 ~ 5 days", "6 ~ 9 days", "10 ~ 19 days" and "more than 20 days". "no" was defined as no drinking behavior, and "1 day" and more than 20 days were defined as drinking behavior.

(5) Screen time: "During your study days (from Monday through Friday), how much time do you spend watching videos or computer games, or doing things on your computer that are not related to study? (Count how much time you spend playing games, watching videos, texting or using social media on your TV, smartphone, computer, iPad or other tablet) "; "On your days off (Saturday through Sunday), how much time do you spend each day watching videos or watching computer games, or doing things on the computer that aren't related to studying? (Count how much time you spend playing games, watching videos, texting or using social media on your TV, smartphone, computer, iPad or other tablet)". Weekday ST and weekend ST include "almost none", "less than 1 hour a day", "less than 2 hours a day", "less than 3 hours a day", "less than 4 hours a day" and "more than 4 hours". The no ST on weekday is defined as the no ST, and have ST is defined as the time with screen. Less than 1 hour a day on rest days was defined as short ST, ≥ 1 hour was defined as long ST.

(6) Skipping breakfast or dinner: "How many days in the last 7 days have you eaten breakfast?" Less than 7 days was defined as insufficient breakfast intake, and 7 days was defined as adequate breakfast intake.

(7) High consumption of fast food/takeaway: "How many times have you eaten takeaway/fast food (such as Ele. me, Humming bird, Meituan takeaway, Baidu takeaway and other fast food) in the last week"; the answers include "have not eaten", "1 ~ 2 times", "3 ~ 4 times" and "5 times or more". "Have not eaten" is defined as no takeaway/fast food consumption, and "have eaten once" or more is defined as takeaway/fast food consumption.

(8) Sugar sweetened beverages (SSBs): "In the last month, you usually drank more than 250 ml (a can) of soft drinks (such as Coke, Sprite, Fanta, etc.) several times a week"; "No" was defined as SSB consumption, and "once" or more was defined as SSBs consumption.

(9) Suicidal ideation (SI), suicidal planning (SP) and suicidal attempt (SA): "Have you seriously considered suicide in the last year?" "Have you made a plan to commit suicide in the past year?" And "Have you committed suicide in the past year?" The answers included "no", "1 time", "2 ~ 3 times" and "4 or more times". No SI, SP and SA were all defined as "no", and have SI, SP and SA were all defined as "1 time" or more.

(10) Non-suicidal self-injury (NSSI): "In the last 12 months, have you ever intentionally hurt yourself without the intention of killing yourself?" The article listed several methods of NSSI: hitting yourself with the fist or palm, pulling your hair, hitting hard objects with your head or fist, pinching or scratching yourself, biting yourself, cutting yourself or stabbing yourself. For those who have hurt themselves, ask them how often they hurt themselves. The frequency of each NSSI is the total number of NSSI [Wan et al., 2019]. "no" is defined as no NSSI, and "1" or more is defined as having NSSI.

*2.5 Data Analysis*

2.5.1 Introduction to research methods of polygenic risk score

The effect of a single SNP locus on multifactorial diseases is often weak, so it is difficult to fully reflect the true association between gene variation and outcome variables in a single mutation or single gene study, while the polygenic risk score (PRS) method can better reflect the association between overall gene variation and outcome variables. PRS is an important method in the study of polygenic diseases. It integrates the comprehensive variable of the variation information of a single SNP in a gene and reflects the overall variation information of the gene [54, 55], especially when the gene score contains many common variations with small effects, its application is more common [56]. Even when a single genetic variation has little effect or no effect, PRS can explain a certain proportion of the variation associated with risk factors and diseases, which makes PRS a very popular method for genetic association research [56]. We recoded the genetic polymorphisms variable as ordinal (homozygous major allele = 0, heterozygous genotype = 1, homozygous minor allele = 2) [56d].

2.5.2 The calculation of PRS

PRS can be divided into unweighted and weighted PRS. Unweighted PRS is obtained by directly adding up the number of mutant alleles in the loci that construct PRS [54]. Weighted PRS is obtained by the product of the number of mutant alleles and the corresponding weight of the gene loci constructing PRS [57,58]. The weight was calculated according to the genetic effect of each allele on the outcome variable, while the genetic effect of each allele on the outcome variable was calculated by Logistic regression with the outcome variable as the dependent variable and each SNP as the independent variable [59].

The specific calculation formula is as follows: unweighted PRS = SNP_l_ + SNP_2_ +... + SNP_i_, weighted PRS = (W_1_ × SNP_l_ + W_2_ × SNP_2_ +... + W_i_ × SNP_i_)/(W_1_ + W_2_ +... + W_i_); Where, i is the number of SNP loci involved in PRS construction, the value of SNP_i_ depends on the number of mutant alleles in gene loci, represented by 0, 1and 2, respectively, and W_i_ is the weight of each gene loci. Unweighted PRS was used in this study.

PRS is obtained by directly adding up the number of mutant alleles in the loci that construct PRS (Burgess & Thompson, 2013). The specific calculation formula is as follows: PRS = SNP_l_ + SNP_2_ +...+ SNP_i_, where i is the number of SNP loci involved in PRS construction, and the value of SNP_i_ depends on the number of mutant alleles in the gene loci, represented by 0, 1, and 2, respectively. The sum of all health risk behavior alleles was used to calculate PRS. In the present study, the PRS ranged from 0 to 98. A higher PRS indicates a higher genetic predisposition to health risk behaviors. PRSs were then classified into three categories according to tertiles: low-risk (< P_33_), medium-risk (≥ P_33_–P_67_), and high-risk (≥ P_67_) groups.

2.5.3 Interaction analysis

In multivariate statistical analysis, interaction refers to the effect of a specific factor on the outcome variable is affected by other factors. In other words, the interaction exists when the effect of two factors on the outcome variable is not equal to the combined effect of the two factors alone [56, 58].

2.5.4 Descriptive statistical analysis

Questionnaire data entry and association analysis were the same as study 2. Counting data were represented by percentile (%), measurement data were represented by mean plus or minus standard deviation(‾*x±s*); independent sample t test was used between the two groups, and one-way analysis of variance (ANOVA) was used for multi-group analysis to compare scores of chronotype of adolescents with different demographic characteristics. Chi-2 test was used to analyze the distribution of clustering of HRB in adolescents with different population characteristics.

*2.7 Sensitivity analysis*

In this study, sensitivity analysis was used to test the robustness of the model: (1) model 1 did not control for covariates; model 2 controlled for gender and age; model 3 controlled for gender, age, parental education level, family residence, only child, self-rated family economic status, number of friends and academic performance; (2) Logistic regression was performed for chronotype with clustering of HRB; (3) Explore the correlation between chronotype and co-occurrence of HRB, and calculate the co-occurrence of HRB using the following methods: to physical activity, smoking, drinking, working day and day off screen time, takeaways/fast food, SSBs, breakfast, vegetables, fruits, the NSSI and suicide ideation, suicide planning and suicide attempt of 14 kinds of actions carried out in accordance with the presence of risk classification, divided into two, according to standards according to the variable will occur for each HRB danger, for 1 minute, the number of all HRB in each research object was added up to form a "co-occurrence index" [60], which divided the HRB co-occurrence into 0, 1-3 and 4 or more types.

**Results**

**3.1 Identification of key factors of HRBs based on multi-source data**

According to the clustering results of HRB risk factor research cataloguing information, **Figure S1 (A)** conducts clustering of information fields, showing four prominent areas: the red areas mainly focus on "students", "violence", "suicidal behavior", "adolescent risk behavior" and "health problems" and “high school student” and “gene”, the yellow area mainly centers on "behavioral risk factor surveil", "binge drinking", "behavior risk factor" and “chronic disease”, the blue area mainly revolves around "drug use", "sexual behavior", "high risk behavior", the green area is dominated by "cardiovascular disease", "mortality" and "physical activity". **Figure S1 (B)** shows the visualization result of project density, whose project distribution is consistent with network visualization. Each node in the project density visualization has a color that indicates the density of the project on that node. The colors range from blue to green to red. The more items near a node, the higher the weight of adjacent items, the more red the color of the node. Conversely, the fewer the number of items near a node, the lower the weight of adjacent items, the closer the color of the node is to blue. **Figure S1 (C)** shows the same item for overlay visualization as for web visualization, the difference being the color of the item. The colors of the projects range from blue to green to red, from far to near, corresponding to the year of the project cluster. According to the trend of node color changes in the figure, researchers' orientation shifted from sexual behaviors and communicable diseases to suicidal, NSSI, sedentary behaviors and psychological problems, etc.

**3.7 Genetic distribution of adolescent clustering of HRBs**

First, according to the results of GWAS and the method of P value < 5×10^−8^, among the results of clustering of HRBs and HRB co-occurrence, only part of the SNP sites of PROK2 gene met the requirements. Second, 372 genes were screened from clustering of HRBs according to P value < 5×10^−4^ (**Figure S3-4**). Similarly results among HRB co-occurrence were shown in **Figure S5-6**. Thirdly, NPAS2 (rs13025524, rs3768984, rs11673746) and ARNTL (rs2278749) meet the requirements of P < 0.05. According to previous references, in the final candidate gene analysis, 49 SNPs were included in 9 candidate genes including CLOCK, ARNTL, NPAS2, PER1, PER2, PER3, CRY1, CRY2 and NR1D1, and the SNPs information of each gene is shown in **Table S13**. **Table S13** also shows the differences between TREND and ALLELIC models for alleles as well as the dominant model (Dom) and recessive model (Rec).

**Table** **supplement**

| Table S1 Screening results of latent categories of health risk behaviors | | | | | | |
| --- | --- | --- | --- | --- | --- | --- |
|  | AIC | BIC | aBIC | LMR-LRT | BLRT | Entropy |
| 1 | 3993.55 | 4043.61 | 3999.23 |  |  |  |
| 2 | 3856.74 | 3960.44 | 3868.50 | ＜0.001 | ＜0.001 | 0.872 |
| 3 | 3825.15 | 3982.49 | 3842.99 | 0.1478 | ＜0.001 | 0.706 |

| Tab. S2 The distribution of demographic characteristics on clustering of HRBs(n＝264) | | | | |
| --- | --- | --- | --- | --- |
| Variables | Total N(%) | High n(%) | Low n(%) | *χ*^2^ value |
| Age | 264 | 13.93±1.66 | 14.26±1.92 | 1.08 |
| Gender |  |  |  | 0.03 |
| Male | 164(62.1) | 30(18.3) | 134(81.7) |  |
| Female | 100(37.9) | 19(19.0) | 81(81.0) |  |
| Residential areas |  |  |  | 2.34 |
| Country | 16(6.1) | 3(18.8) | 13(81.3) |  |
| Town | 42(15.8) | 4(9.5) | 38(90.5) |  |
| Urban | 206(78.1) | 40(19.4) | 166(80.6) |  |
| Only child |  |  |  | 0.01 |
| Yes | 69(26.1) | 12(17.4) | 57(82.6) |  |
| No | 195(73.9) | 35(17.9) | 160(82.1) |  |
| Father's education |  |  |  | 0.12 |
| Junior high and below | 95(36.0) | 16(16.8) | 79(83.2) |  |
| High school or technical secondary school | 96(36.4) | 18(18.8) | 78(81.3) |  |
| Junior college or above | 73(27.7) | 13(17.8) | 60(82.2) |  |
| Mother's education |  |  |  | 2.08 |
| Junior high and below | 118(44.7) | 20(17.1) | 98(82.9) |  |
| High school or technical secondary school | 86(32.6) | 13(14.9) | 74(85.1) |  |
| Junior college or above | 60(22.7) | 14(23.3) | 46(76.7) |  |
| Family economic conditions |  |  |  | 3.11 |
| Very bad | 5(1.9) | 1(20.0) | 4(80.0) |  |
| Worse | 19(7.2) | 6(31.6) | 13(68.4) |  |
| Medium | 181(68.6) | 31(17.1) | 150(82.9) |  |
| Better | 44(16.7) | 6(13.6) | 38(86.4) |  |
| Very good | 15(5.7) | 3(20.0) | 12(80.0) |  |
| Friends number |  |  |  | 1.11 |
| No | 9(3.4) | 1(11.1) | 8(88.9) |  |
| 1-2 | 49(18.6) | 9(18.4) | 40(81.6) |  |
| 3-5 | 86(32.6) | 13(15.1) | 73(84.9) |  |
| 6 or more | 120(45.5) | 24(20.0) | 96(80.0) |  |
| Learning burden |  |  |  | 2.26 |
| Light | 18(6.8) | 5(27.8) | 13(72.2) |  |
| Medium | 157(59.5) | 24(15.3) | 133(84.7) |  |
| Heavy | 89(33.6) | 18(20.2) | 71(79.8) |  |

| Table S3 The relationship between mental health and clustering of HRBs in adolescents | | | |
| --- | --- | --- | --- |
| Mental health | High | Low | P value |
| Psychological sub-health | 43.69±3.62 | 25.07±1.07 | **＜0.01** |
| Anxiety | 8.09±1.08 | 2.95±0.34 | **＜0.01** |
| Depression | 8.87±1.21 | 4.23±0.44 | **＜0.01** |

| Table S4 The relationship between mental health and HRB co-occurrence in adolescents | | | |
| --- | --- | --- | --- |
| Mental health | High | Low | *P value* |
| Psychological sub-health | 31.94±20.62 | 25.31±16.64 | **＜0.01** |
| Anxiety | 4.93±6.23 | 3.01±5.45 | **＜0.01** |
| Depression | 6.10±7.24 | 4.21±6.71 | ＞0.05 |

| Tab. S5 Association between chronotype and HRB co-occurrence in adolescents | | | | | | | |
| --- | --- | --- | --- | --- | --- | --- | --- |
| Variables | Total model | |  | Male | | Female | |
|  | Low | High |  | Low | High | Low | High |
| Chronotype |  |  |  |  |  |  |  |
| Eveningness | 1.0 | **2.41(1.02,5.71)*** |  | 1.0 | **5.38(1.42,20.38)*** | 1.0 | 1.21(0.35,4.20) |
| Intermidiate | 1.0 | 1.51(0.84,2.69) |  | 1.0 | 2.0(0.91,1.39) | 1.0 | 1.04(0.39,2.77) |
| Morningness | 1.0 | 1.0 |  | 1.0 | 1.0 | 1.0 | 1.0 |
| Total model were adjusted for gender, age, educational level of parents, family residence, only child, self-rated family financial status, number of friends and learning burden | | | | | | | |

| Tab. S6 Association between chronotype and psychological sub-health in adolescents | | | | | | | |
| --- | --- | --- | --- | --- | --- | --- | --- |
| Variables | Total model | |  | Male | | Female | |
|  | Low | High |  | Low | High | Low | High |
| Chronotype |  |  |  |  |  |  |  |
| Eveningness | 1.0 | 1.52(0.42,5.50) |  | 1.0 | 1.43(0.13,10.10) | 1.0 | 0.93(0.14,6.45) |
| Intermidiate | 1.0 | 0.90(0.34,2.36) |  | 1.0 | 0.52(0.12,2.22) | 1.0 | 0.63(0.12,3.43) |
| Morningness | 1.0 | 1.0 |  | 1.0 | 1.0 | 1.0 | 1.0 |
| Total model were adjusted for gender, age, educational level of parents, family residence, only child, self-rated family financial status, number of friends and learning burden | | | | | | | |

| Tab. S7 Association between chronotype and depression in adolescents | | | | | | | |
| --- | --- | --- | --- | --- | --- | --- | --- |
| Variables | Total model | |  | Male | | Female | |
|  | Low | High |  | Low | High | Low | High |
| Chronotype |  |  |  |  |  |  |  |
| Eveningness | 1.0 | **2.65(1.10,6.39)*** |  | 1.0 | 1.07(0.28,4.14) | 1.0 | **4.52(1.29,18.75)*** |
| Intermidiate | 1.0 | 1.57(0.83,2.95) |  | 1.0 | 1.20(0.53,2.70) | 1.0 | 1.72(0.59,4.97) |
| Morningness | 1.0 | 1.0 |  | 1.0 | 1.0 | 1.0 | 1.0 |
| Total model were adjusted for gender, age, educational level of parents, family residence, only child, self-rated family financial status, number of friends and learning burden | | | | | | | |

| Tab. S8 Association between chronotype and anxiety in adolescents | | | | | | | |
| --- | --- | --- | --- | --- | --- | --- | --- |
| Variables | Total model | |  | Male | | Female | |
|  | Low | High |  | Low | High | Low | High |
| Chronotype |  |  |  |  |  |  |  |
| Eveningness | 1.0 | 1.01(0.39,2.62) |  | 1.0 | 0.95(0.22,4.24) | 1.0 | 1.25(0.29,5.42) |
| Intermidiate | 1.0 | 1.48(0.78,2.82) |  | 1.0 | 1.39(0.59,3.25) | 1.0 | 1.99(0.63,6.30) |
| Morningness | 1.0 | 1.0 |  | 1.0 | 1.0 | 1.0 | 1.0 |
| Total model were adjusted for gender, age, educational level of parents, family residence, only child, self-rated family financial status, number of friends and learning burden | | | | | | | |

| Table S9 Testing the moderated mediation effects of chronotype and depression on the HRB co-occurrence among adolescents | | | | | | |
| --- | --- | --- | --- | --- | --- | --- |
| Variables | PHQ | | | HRB co-occurrence | | |
|  | B | t value | *P* value | B | t value | *P* value |
| Chronotype(totalE1) | 0.29 | 0.84 | ＞0.05 | -0.31 | -3.08 | **＜0.01** |
| Gender | 5.31 | 1.40 | ＞0.05 | -2.46 | -2.18 | **＜0.05** |
| Gender*Chronotype | -0.31 | -1.27 | ＞0.05 | 0.17 | 2.41 | **＜0.05** |
| PHQ(total) |  |  |  | -0.06 | -1.12 | ＞0.05 |
| PHQ*gender |  |  |  | 0.075 | 1.99 | **＜0.05** |
| R^2^ | 0.014 | | | 0.09 | | |
| F | 1.19 | | | 5.33 | | |
| Mediate variables: HRB, moderated variables: MEQ, independent variables: SERF, dependent variables: mental health symptoms. The model was controlled for age, gender, grade, parental education, family economic status, numbers of friends, residential areas, academic record. | | | | | | |

| Table S10 Bootstrapped conditional direct and indirect effects between depression and HRB co-occurrence | | | | | |
| --- | --- | --- | --- | --- | --- |
|  |  | | HRB co-occurrence | | |
| Direct effect |  |  | Effect | SE | (LL,UL) |
|  | Predictor | Chronotype |  |  |  |
|  | Moderator (gender) | Male | -0.1433 | 0.0434 | -0.2288,-0.0577 |
|  |  | Female | -0.0061 | 0.0554 | -0.1153,0.1030 |
| Indirect effect |  |  | Effect | SE | (LL,UL) |
|  | Predictor | HRB |  |  |  |
|  | Mediator (GAD) | Low | -0.0006 | 0.0061 | -0.0201,0.0073 |
|  |  | High | -0.0030 | 0.0212 | -0.0463,0.0434 |

| Table S11 Testing the moderated mediation effects of chronotype and anxiety on the HRB co-occurrence among adolescents | | | | | | |
| --- | --- | --- | --- | --- | --- | --- |
| Variables | GAD | | | HRB co-occurrence | | |
|  | B | t value | *P* value | B | t value | *P* value |
| Chronotype(totalE1) | 1.0 | 0.56 | ＞0.05 | -1.83 | -3.10 | **＜0.01** |
| Gender | 1.72 | 0.66 | ＞0.05 | -1.78 | -2.03 | **＜0.05** |
| Gender*Chronotype | -0.57 | -0.49 | ＞0.05 | 0.88 | 2.28 | **＜0.05** |
| GAD(total) |  |  |  | -0.06 | -0.97 | ＞0.05 |
| GAD*gender |  |  |  | 0.092 | 2.17 | **＜0.05** |
| R^2^ | 0.0028 | | | 0.12 | | |
| F | 0.24 | | | 6.93 | | |
| Mediate variables: HRB, moderated variables: MEQ, independent variables: SERF, dependent variables: mental health symptoms. The model was controlled for age, gender, grade, parental education, family economic status, numbers of friends, residential areas, academic record. | | | | | | |

| Table S12 Bootstrapped conditional direct and indirect effects between anxiety and HRB co-occurrence | | | | | |
| --- | --- | --- | --- | --- | --- |
|  |  | | HRB co-occurrence | | |
| Direct effect |  |  | Effect | SE | (LL,UL) |
|  | Predictor | Chronotype |  |  |  |
|  | Moderator (gender) | Male | -0.95 | 0.26 | -1.45, -0.44 |
|  |  | Female | -0.06 | 0.29 | -0.63, 0.51 |
| Indirect effect |  |  | Effect | SE | (LL,UL) |
|  | Predictor | HRB |  |  |  |
|  | Mediator (GAD) | Low | 0.013 | 0.035 | -0.023, 0.14 |
|  |  | High | -0.018 | 0.12 | -0.28, 0.20 |

| Tab. S13 The allele and genotype frequencies of CLOCK gene polymorphisms in clustering of health risk behavior among adolescents | | | | | | | | | | | | | |
| --- | --- | --- | --- | --- | --- | --- | --- | --- | --- | --- | --- | --- | --- |
| **Chr** | **POS** | **SNP** | **gene** | **minor** | **major** | **Genotype**  **(high level)** | **Genotype**  **(low level)** | ***P-*value**  **(fisher)** | **P for trend** | **Geno** | **Dom** | **Rec** | **Function** |
| chr11 | 13280374 | rs1481892 | *ARNTL* | G | C | 5/25/17 | 47/105/59 | 0.08652 | 0.07804 | 0.1554 | 0.2901 | 0.1058 | intronic |
| chr11 | 13285058 | rs4146388 | *ARNTL* | T | C | 6/25/16 | 46/105/60 | 0.2093 | 0.1936 | 0.3616 | 0.4807 | 0.2269 | intronic |
| chr11 | 13291422 | rs7107287 | *ARNTL* | T | G | 6/24/17 | 46/105/60 | 0.1689 | 0.1377 | 0.3063 | 0.2958 | 0.2269 | intronic |
| chr11 | 13352217 | rs11022775 | *ARNTL* | T | C | 0/12/35 | 4/29/178 | 0.2437 | 0.2489 | 0.1185 | 0.1351 | 1 | intronic |
| chr11 | 13362135 | rs1868049 | *ARNTL* | C | T | 10/27/10 | 53/103/55 | 1 | 0.9335 | 0.6144 | 0.5797 | 0.708 | intronic |
| chr11 | 13363769 | rs3789327 | *ARNTL* | G | A | 3/24/20 | 24/86/101 | 1 | 0.976 | 0.3811 | 0.5232 | 0.4324 | intronic |
| chr11 | 13375362 | rs11022780 | *ARNTL* | T | C | 6/18/23 | 15/93/103 | 0.6182 | 0.5893 | 0.3899 | 1 | 0.2349 | intronic |
| chr11 | 13376331 | rs2278749 | *ARNTL* | T | C | 1/6/40 | 6/64/141 | **0.02945** | **0.0244** | **0.03296** | **0.01343** | 1 | intronic |
| chr11 | 13276938 | rs2279287 | *ARNTL* | T | C | 5/25/17 | 47/104/60 | 0.1083 | 0.08642 | 0.1642 | 0.2958 | 0.1058 | upstream |
| chr4 | 55432133 | rs10462028 | *CLOCK* | A | G | 0/5/42 | 1/22/188 | 1 | 0.8893 | 1 | 1 | 1 | UTR3 |
| chr4 | 55435202 | rs1801260 | *CLOCK* | G | A | 0/5/42 | 1/22/188 | 1 | 0.8893 | 1 | 1 | 1 | UTR3 |
| chr4 | 55440643 | rs3805148 | *CLOCK* | A | C | 3/19/25 | 26/91/94 | 0.1828 | 0.1787 | 0.4369 | 0.3324 | 0.3135 | intronic |
| chr4 | 55443825 | rs3736544 | *CLOCK* | A | G | 2/16/29 | 18/83/110 | 0.199 | 0.1766 | 0.4574 | 0.2601 | 0.5449 | synonymous |
| chr4 | 55452295 | rs6849474 | *CLOCK* | G | A | 3/19/25 | 25/92/94 | 0.2229 | 0.19 | 0.4439 | 0.3324 | 0.4353 | intronic |
| chr4 | 55454900 | rs2412648 | *CLOCK* | T | G | 3/19/25 | 25/92/94 | 0.2229 | 0.19 | 0.4439 | 0.3324 | 0.4353 | intronic |
| chr4 | 55460485 | rs11133385 | *CLOCK* | G | A | 3/19/25 | 25/92/94 | 0.2229 | 0.19 | 0.4439 | 0.3324 | 0.4353 | intronic |
| chr4 | 55462689 | rs4340844 | *CLOCK* | A | C | 3/19/25 | 25/92/94 | 0.2229 | 0.19 | 0.4439 | 0.3324 | 0.4353 | intronic |
| chr4 | 55482360 | rs12504300 | *CLOCK* | G | C | 3/19/25 | 25/92/94 | 0.2229 | 0.19 | 0.4439 | 0.3324 | 0.4353 | intronic |
| chr4 | 55487920 | rs4864542 | *CLOCK* | C | G | 3/19/25 | 25/92/94 | 0.2229 | 0.19 | 0.4439 | 0.3324 | 0.4353 | intronic |
| chr4 | 55492116 | rs11939815 | *CLOCK* | T | G | 2/16/29 | 17/84/110 | 0.1998 | 0.1878 | 0.4662 | 0.2601 | 0.5407 | intronic |
| chr4 | 55501955 | rs12648271 | *CLOCK* | G | C | 3/19/25 | 25/92/94 | 0.2229 | 0.19 | 0.4439 | 0.3324 | 0.4353 | intronic |
| chr4 | 55514317 | rs12649507 | *CLOCK* | G | A | 3/19/25 | 25/92/94 | 0.2229 | 0.19 | 0.4439 | 0.3324 | 0.4353 | intronic |
| chr4 | 55515830 | rs6850524 | *CLOCK* | C | G | 2/16/29 | 17/84/110 | 0.1998 | 0.1878 | 0.4662 | 0.2601 | 0.5407 | intronic |
| chr4 | 55526646 | rs13132420 | *CLOCK* | G | A | 3/19/25 | 25/92/94 | 0.2229 | 0.19 | 0.4439 | 0.3324 | 0.4353 | intronic |
| chr4 | 55547664 | rs11133399 | *CLOCK* | G | A | 2/16/29 | 17/84/110 | 0.1998 | 0.1878 | 0.4662 | 0.2601 | 0.5407 | upstream |
| chr12 | 107093085 | rs3809238 | *CRY1* | A | G | 4/16/27 | 10/66/135 | 0.2681 | 0.2823 | 0.4196 | 0.4089 | 0.2931 | UTR5 |
| chr11 | 45856137 | rs2292912 | *CRY2* | G | C | 4/18/25 | 23/101/87 | 0.2268 | 0.175 | 0.3695 | 0.1457 | 0.7948 | intronic |
| chr11 | 45882062 | rs2292910 | *CRY2* | C | A | 12/20/5 | 15/90/106 | 0.3721 | 0.3254 | 0.0755 | 0.107 | 0.378 | UTR3 |
| chr2 | 100862552 | rs1811399 | *NPAS2* | C | A | 5/21/21 | 24/98/89 | 0.8109 | 0.7623 | 0.9727 | 0.7477 | 1 | intronic |
| chr2 | 100977729 | rs11541353 | *NPAS2* | T | C | 0/1/46 | 0/1/210 | 0.3314 | 0.2424 | 0.3317 | 0.3317 | 1 | missense |
| chr2 | 100987350 | rs11123857 | *NPAS2* | G | A | 0/1/46 | 0/2/209 | 0.4537 | 0.495 | 0.4544 | 0.4544 | 1 | intronic |
| chr2 | 100996106 | rs3739008 | *NPAS2* | T | C | 1/17/29 | 11/76/124 | 0.5875 | 0.5226 | 0.8188 | 0.7454 | 0.7004 | UTR3 |
| chr2 | 100933719 | rs13025524 | *NPAS2* | A | G | 8/23/16 | 13/75/123 | **0.0008161** | **0.00068** | **0.003059** | **0.003371** | 0.03282 | intronic |
| chr2 | 100934525 | rs3768984 | *NPAS2* | C | A | 4/14/29 | 23/105/83 | **0.02192** | **0.01929** | **0.02059** | **0.005832** | 0.7948 | intronic |
| chr2 | 100952681 | rs11673746 | *NPAS2* | T | C | 1/7/39 | 0/15/196 | **0.02515** | **0.01392** | **0.02991** | **0.04467** | 0.1822 | intronic |
| chr2 | 100968433 | rs2289950 | *NPAS2* | T | C | 12/20/15 | 34/105/72 | 0.3016 | 0.3033 | 0.3072 | 0.8652 | 0.1415 | intronic |
| chr17 | 40096959 | rs2314339 | *NR1D1* | C | T | 9/29/9 | 59/85/67 | 0.8196 | 0.7528 | 0.03038 | 0.1109 | 0.2727 | intronic |
| chr17 | 40098239 | rs2071427 | *NR1D1* | T | C | 8/23/16 | 50/96/65 | 0.4235 | 0.399 | 0.6368 | 0.7287 | 0.4395 | intronic |
| chr17 | 40098436 | rs2269457 | *NR1D1* | C | T | 6/26/15 | 45/99/67 | 0.4911 | 0.4449 | 0.3914 | 1 | 0.2265 | intronic |
| chr17 | 40100440 | rs939347 | *NR1D1* | A | G | 6/29/12 | 43/112/56 | 0.5699 | 0.5407 | 0.4879 | 1 | 0.3044 | UTR5 |
| chr17 | 8145667 | rs3027188 | *PER1* | C | G | 3/23/21 | 24/95/92 | 0.6293 | 0.5667 | 0.6557 | 1 | 0.4324 | intronic |
| chr17 | 8152405 | rs3027172 | *PER1* | G | A | 0/6/41 | 1/16/194 | 0.4143 | 0.387 | 0.3902 | 0.3927 | 1 | UTR5 |
| chr17 | 8153288 | rs2518023 | *PER1* | T | G | 12/22/13 | 38/115/58 | 0.5676 | 0.5027 | 0.4483 | 1 | 0.3065 | upstream |
| chr2 | 238246412 | rs934945 | *PER2* | T | C | 3/20/24 | 24/80/107 | 0.7086 | 0.6213 | 0.635 | 1 | 0.4324 | missense |
| chr2 | 238257022 | rs2304669 | *PER2* | C | T | 0/11/36 | 2/40/169 | 0.7127 | 0.7118 | 0.6961 | 0.5565 | 1 | synonymous |
| chr2 | 238277948 | rs2304672 | *PER2* | C | G | 0/5/42 | 1/17/193 | 0.7858 | 0.7386 | 0.646 | 0.5818 | 1 | UTR5 |
| chr1 | 7787776 | rs228727 | *PER3* | T | C | 9/27/11 | 35/99/77 | 0.167 | 0.1625 | 0.2261 | 0.09211 | 0.6706 | intronic |
| chr1 | 7798075 | rs1012477 | *PER3* | C | G | 0/2/45 | 1/13/197 | 0.7498 | 0.5015 | 1 | 0.7439 | 1 | intronic |
| chr1 | 7809988 | rs228669 | *PER3* | T | C | 1/22/24 | 15/88/108 | 0.7024 | 0.621 | 0.4794 | 1 | 0.3185 | synonymous |

| Tab. S14 Subgroup analysis of the association between CLOCK-PRS and adolescent clustering of HRB | | | | | | | | | |
| --- | --- | --- | --- | --- | --- | --- | --- | --- | --- |
| Variables | Clustering of HRBs | | | | | | | | |
|  | *coeff* | *SE* | | | *t* value | | *P* value | LLCI | ULCI |
| PRS | 0.24 | | 0.11 | 2.24 | | **0.02** | | 0.03 | 0.46 |
| Only child | 7.14 | | 3.29 | 2.17 | | **0.03** | | 0.69 | 13.59 |
| Int_1 | -0.12 | | 0.06 | -2.17 | | **0.03** | | -0.23 | -0.01 |
| Int_1: PRS×only child；Adjusted for gender, age, educational level of parents, residential areas, only child, self-rated family financial status, number of friends and learning burden | | | | | | | | | |

| Tab. S15 Association of CLOCK-PRS and chronotype interactions with anxiety in adolescents | | | | | | | |
| --- | --- | --- | --- | --- | --- | --- | --- |
| Variables | Model 1 | |  | Model 2 | | Model 3 | |
|  | Low | High |  | Low | High | Low | High |
| PRS+chronotype |  |  |  |  |  |  |  |
| High PRS＋eveningness | 1.0 | 1.09(0.17,6.94) |  | 1.0 | 0.98(0.15,6.34) | 1.0 | 0.98(0.14,6.63) |
| High PRS＋intermidiate | 1.0 | 1.41(0.44,4.56) |  | 1.0 | 1.45(0.45,4.730 | 1.0 | 1.80(0.52,6.29) |
| High PRS＋morningness | 1.0 | 1.27(0.34,4.670 |  | 1.0 | 1.33(0.36,4.95) | 1.0 | 1.51(0.37,6.20) |
| Medium PRS＋eveningness | 1.0 | 0.48(0.05,4.74) |  | 1.0 | 0.47(0.04,4.32) | 1.0 | 0.52(0.05,6.0) |
| Medium PRS＋intermidiate | 1.0 | 1.73(0.54,5.500 |  | 1.0 | 1.65(0.52,5.30) | 1.0 | 2.16(0.62,7.52) |
| Medium PRS＋morningness | 1.0 | 1.34(0.35,5.20) |  | 1.0 | 1.41(0.36,5.49) | 1.0 | 1.81(0.42,7.68) |
| Low PRS＋eveningness | 1.0 | 3.8(0.90,16.01) |  | 1.0 | 3.45(0.81,14.68) | 1.0 | **5.62(1.17,27.13)^*^** |
| Low PRS＋intermidiate | 1.0 | 2.88(0.93,8.93) |  | 1.0 | **3.20(1.02,10.05)^*^** | 1.0 | **4.61(1.34,15.90)^*^** |
| Low PRS＋morningness | 1.0 | 1.0 |  | 1.0 | 1.0 |  |  |
| Model 1 : crude model ; Model 2: Adjusted for gender, age; Model 2: Adjusted for gender, age, educational level of parents, family residence, only child, self-rated family financial status, number of friends and learning burden | | | | | | | |

| Tab. S16 Association of CLOCK-PRS and chronotype interactions with depression in adolescents | | | | | | | |
| --- | --- | --- | --- | --- | --- | --- | --- |
| Variables | Model 1 | |  | Model 2 | | Model 3 | |
|  | Low | High |  | Low | High | Low | High |
| PRS+chronotype |  |  |  |  |  |  |  |
| High PRS＋eveningness | 1.0 | 3.04(0.62,14.77) |  | 1.0 | 2.72(0.55,13.36) | 1.0 | 2.47(0.48,12.61) |
| High PRS＋intermidiate | 1.0 | 1.46(0.51,4.190 |  | 1.0 | 1.51(0.52,4.39) | 1.0 | 1.84(0.61,5.60) |
| High PRS＋morningness | 1.0 | 1.15(0.35,3.76) |  | 1.0 | 1.22(0.37,4.04) | 1.0 | 1.40(0.39,4.99) |
| Medium PRS＋eveningness | 1.0 | 1.94(0.40,9.45) |  | 1.0 | 1.74(0.35,8.54) | 1.0 | 2.38(0.43,13.28) |
| Medium PRS＋intermidiate | 1.0 | 1.0(0.34,2.940 |  | 1.0 | 0.94(0.32,2.80) | 1.0 | 1.04(0.33,3.28) |
| Medium PRS＋morningness | 1.0 | 0.86(0.24,3.09) |  | 1.0 | 0.90(0.25,3.28) | 1.0 | 1.09(0.28,4.23) |
| Low PRS＋eveningness | 1.0 | **4.37(1.07,17.79)*** |  | 1.0 | 3.91(0.95,16.12) | 1.0 | **5.54(1.23,24.96)*** |
| Low PRS＋intermidiate | 1.0 | 2.16(0.77,6.09) |  | 1.0 | 2.41(0.84,6.92) | 1.0 | **3.13(1.02,9.61)*** |
| Low PRS＋morningness | 1.0 | 1.0 |  | 1.0 | 1.0 |  |  |
| Model 1 : crude model ; Model 2: Adjusted for gender, age; Model 2: Adjusted for gender, age, educational level of parents, family residence, only child, self-rated family financial status, number of friends and learning burden | | | | | | | |

| Table S17 Testing the moderated mediation effects of chronotype and anxiety on the clustering of HRBs among adolescents | | | | | | |
| --- | --- | --- | --- | --- | --- | --- |
| Variables | GAD(total) | | | Clustering of HRBs | | |
|  | B | t value | *P* value | B | t value | *P* value |
| Chronotype(totalE1) | -3.83 | -1.07 | ＞0.05 | -2.07 | -1.15 | ＞0.05 |
| PRS | -0.17 | -1.49 | ＞0.05 | -0.05 | -0.88 | ＞0.05 |
| PRS*Chronotype | 0.059 | 1.11 | ＞0.05 | 0.023 | 0.89 | ＞0.05 |
| GAD(total) |  |  |  | -0.20 | -1.12 | ＞0.05 |
| GAD*PRS |  |  |  | 0.005 | 1.85 | 0.0649 |
| R^2^ | 0.013 | | |  | | |
| F | 1.06 | | |  | | |
| Mediate variables: GAD, moderated variables: PRS, independent variables: MEQ, dependent variables: HRB. The model was controlled for age, gender, grade, parental education, family economic status, numbers of friends, residential areas, academic record. | | | | | | |

| Table S18 Bootstrapped conditional direct and indirect effects between anxiety and clustering of HRBs | | | | | |
| --- | --- | --- | --- | --- | --- |
|  |  | | Clustering of HRB | | |
| Direct effect |  |  | Effect | SE | (LL,UL) |
|  | Predictor | Chronotype |  |  |  |
|  | Moderator (gender) | Low | -0.7461 | 0.3976 | -1.5254, 0.0333 |
|  |  | Medium | -0.5080 | 0.2882 | -1.0728, 0.0568 |
|  |  | High | -0.2700 | 0.3903 | -1.0349, 0.4949 |
| Indirect effect |  |  | Effect | SE | (LL,UL) |
|  | Predictor | HRB |  |  |  |
|  | Mediator (GAD) | Low | 56.9257 | 0.0799 | -0.2506, 0.0956 |
|  |  | Mudium | 67.1417 | 0.0857 | -0.1669, 0.1781 |
|  |  | High | 77.3578 | 0.1495 | -0.1381, 0.4723 |

| Table S19 Testing the moderated mediation effects of chronotype and anxiety on the HRB co-occurrence among adolescents | | | | | | |
| --- | --- | --- | --- | --- | --- | --- |
| Variables | GAD | | | HRB co-occurrence(HRB5) | | |
|  | B | t value | *P* value | B | t value | *P* value |
| Chronotype(totalE1) | -3.83 | -1.07 | ＞0.05 | 1.28 | 0.98 | ＞0.05 |
| PRS(total) | -0.17 | -1.49 | ＞0.05 | 0.036 | 0.80 | ＞0.05 |
| PRS*Chronotype | 0.059 | 1.11 | ＞0.05 | -0.028 | -1.40 | ＞0.05 |
| GAD(total) |  |  |  | -0.29 | -1.90 | ＞0.05 |
| GAD*PRS |  |  |  | 0.005 | 2.29 | **< 0.05** |
| R^2^ | 0.013 | | |  | | |
| F | 1.06 | | |  | | |
| Mediate variables: GAD, moderated variables: PRS, independent variables: MEQ, dependent variables: HRB. The model was controlled for age, gender, grade, parental education, family economic status, numbers of friends, residential areas, academic record. | | | | | | |

| Table S20 Bootstrapped conditional direct and indirect effects bweteen anxiety and HRB co-occurrence | | | | | |
| --- | --- | --- | --- | --- | --- |
|  |  | | HRB co-occurrence(HRB5) | | |
| Direct effect |  |  | Effect | SE | (LL,UL) |
|  | Predictor | Chronotype |  |  |  |
|  | Moderator (gender) | Low | -0.2901 | 0.2779 | -0.8348, 0.2545 |
|  |  | Medium | -0.5722 | 0.2189 | -1.0012, -0.1431 |
|  |  | High | -0.8542 | 0.3157 | -1.4730, 0.2355 |
| Indirect effect |  |  | Effect | SE | (LL,UL) |
|  | Predictor | HRB |  |  |  |
|  | Mediator (GAD) | Low | -0.0072 | 0.0284 | -0.1170, 0.0260 |
|  |  | Mudium | 0.0071 | 0.0454 | -0.0888, 0.1001 |
|  |  | High | 0.0858 | 0.1042 | -0.0910, 0.3335 |

**Figure supplement**


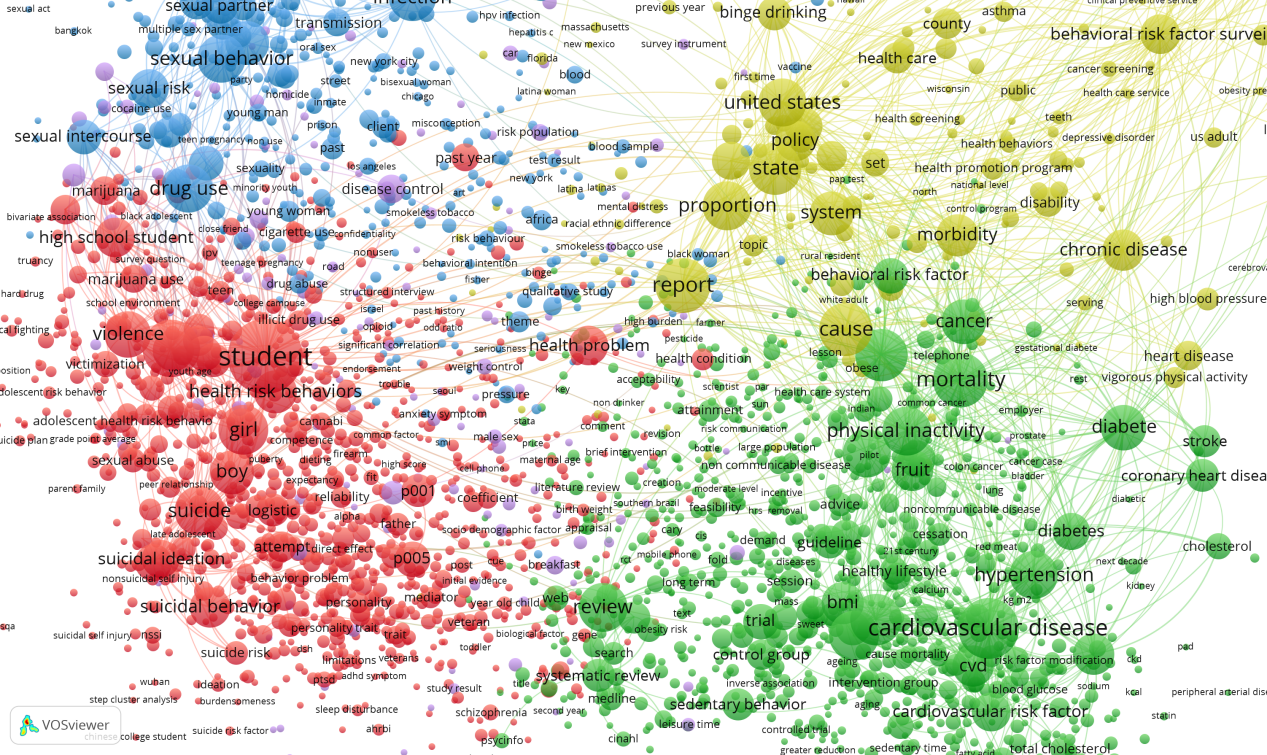


A


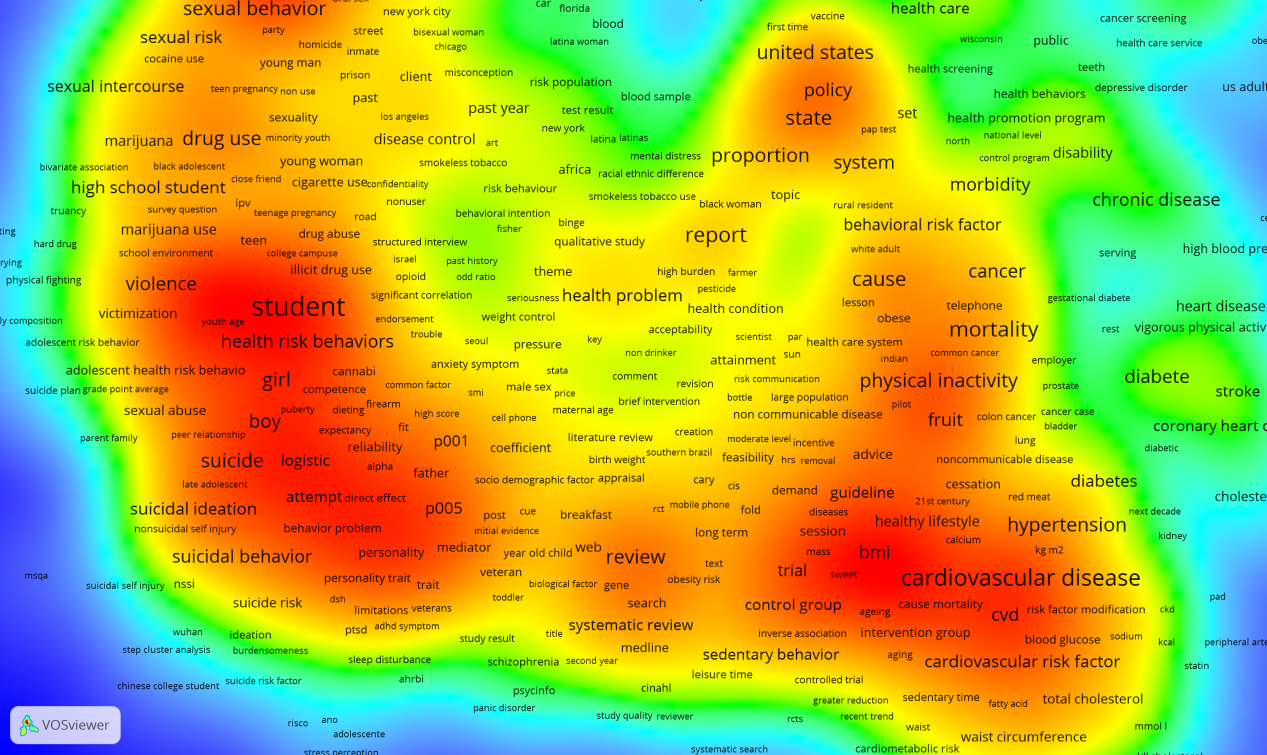


B


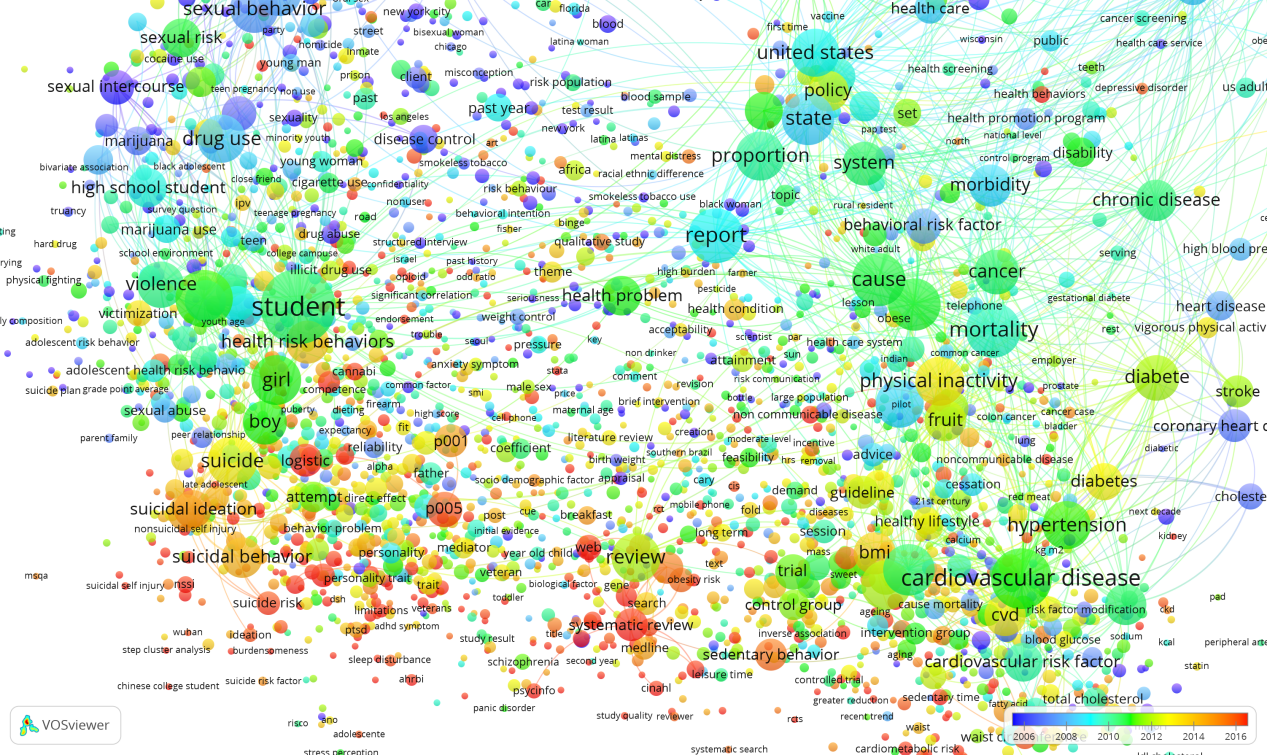


C

**Fig. S1 Health risk factors knowledge domain mapping map (A) Network Visualization (B) Density Visualization (C) Overlay Visualization**

**Fig. S2 Class of HRBs**


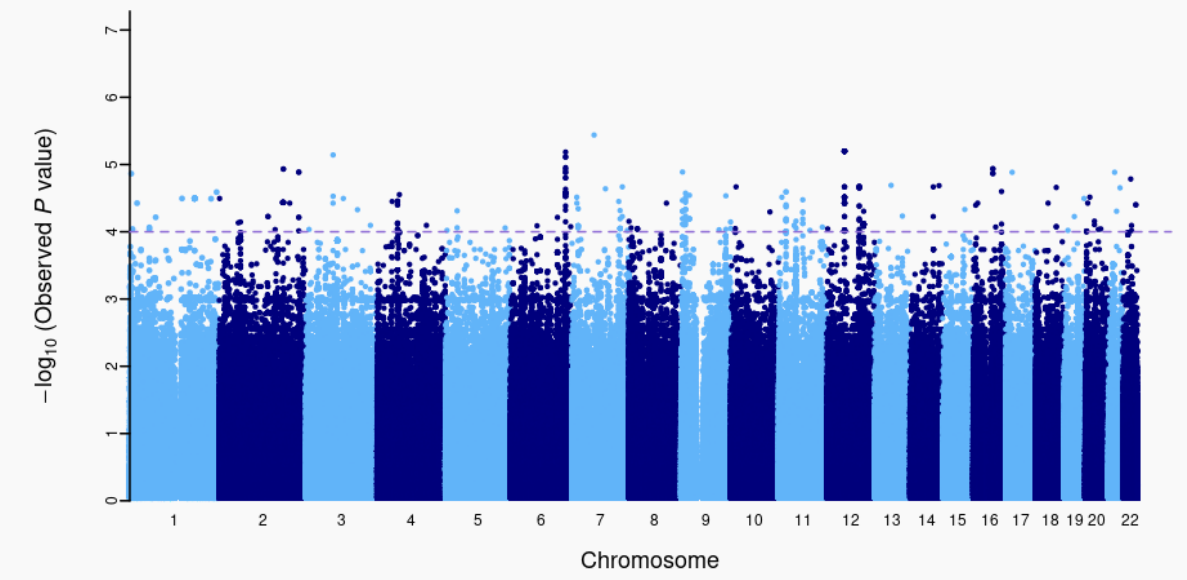


**Fig. S3 Manhattan map of genome-wide association analysis of subjects**

(The horizontal axis represents 22 autochromosomes, the vertical axis represents the -log10 P value of statistical significance, and the horizontal dotted purple line represents the genome-wide significance level of P < 5×10^-4^).


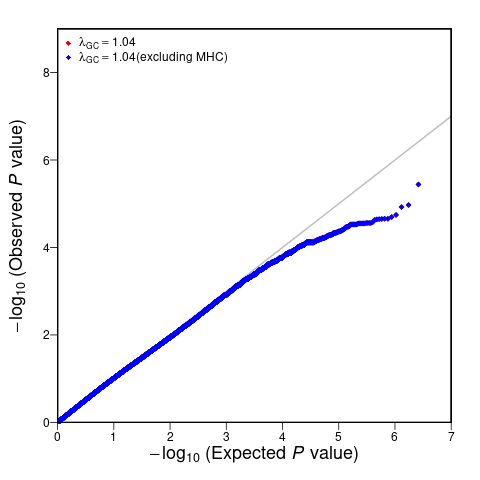


**Fig. S4 Q-Q plot of genome-wide association analysis of subjects**

(The horizontal axis represents the negative logarithm function value of chi-squared expected P value; The Y-axis represents the negative logarithm function value of chi-square measured P value; The gray dots represent SNP sites; The red line is the uncorrelated average under the null hypothesis; λ represents the gene expansion coefficient).


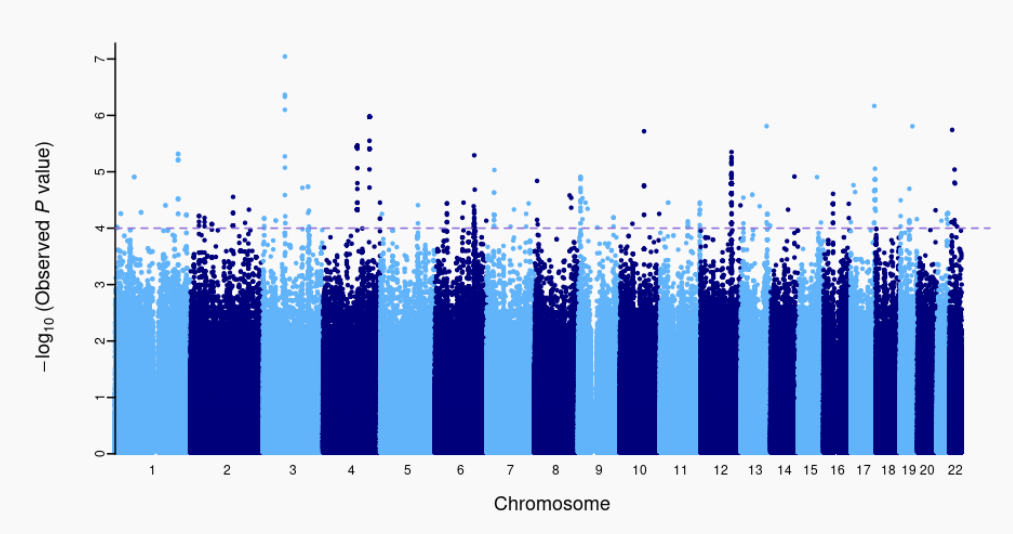


**Fig. S5 Manhattan map of genome-wide association analysis of subjects**

(The horizontal axis represents 22 autochromosomes, the vertical axis represents the -log10 P value of statistical significance, and the horizontal dotted purple line represents the genome-wide significance level of P < 5×10^-4^).


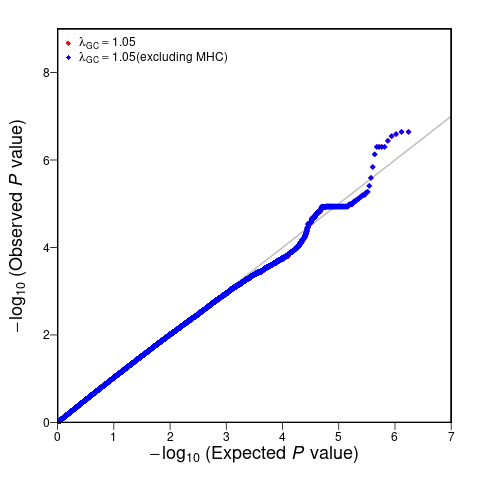


**Fig. S6 Q-Q plot of genome-wide association analysis of subjects**

(The horizontal axis represents the negative logarithm function value of chi-squared expected P value; The Y-axis represents the negative logarithm function value of chi-square measured P value; The gray dots represent SNP sites; The red line is the uncorrelated average under the null hypothesis; λ represents the gene expansion coefficient).
